# Co-delivery of siPTPN13 and siNOX4 *via* (myo)fibroblast-targeting polymeric micelles for idiopathic pulmonary fibrosis therapy

**DOI:** 10.7150/thno.54217

**Published:** 2021-01-09

**Authors:** Jiwei Hou, Qijian Ji, Jie Ji, Shenghong Ju, Chun Xu, Xueqing Yong, Xiaoxuan Xu, Mohd. Muddassir, Xiang Chen, Jinbing Xie, Xiaodong Han

**Affiliations:** 1 Immunology and Reproduction Biology Laboratory & State Key Laboratory of Analytical Chemistry for Life Science, Medical School, Nanjing University, Nanjing, 210093, China.; 2 Jiangsu Key Laboratory of Molecular Medicine, Nanjing University, Nanjing, 210093, China.; 3 Department of Critical Care Medicine, Xuyi People's Hospital, 28 Hongwu Road, Xuyi, 211700, Jiangsu, China.; 4 Department of Emergency Medicine, Jinling Hospital, Medical School of Nanjing University, Nanjing, 210002, PR China.; 5 Jiangsu Key Laboratory of Molecular Imaging and Functional Imaging, Department of Radiology, Zhongda Hospital, Medical School, Southeast University, 87 Dingjiaqiao Road, Nanjing 210009, China.; 6 Department of Pathology, Medical School of Southeast University, 87 Dingjiaqiao, Nanjing, 210009, China.; 7 Department of Nuclear Science & Technology, Nanjing University of Aeronautics and Astronautics, Nanjing, 211106, China.; 8 Department of Chemistry, College of Science, King Saud University, Riyadh 11451, Saudi Arabia.

**Keywords:** idiopathic pulmonary fibrosis (IPF), (myo)fibroblast, activation, apoptosis, siRNA, micelle

## Abstract

**Rationale:** (Myo)fibroblasts are the ultimate effector cells responsible for the production of collagen within alveolar structures, a core phenomenon in the pathogenesis of idiopathic pulmonary fibrosis (IPF). Although (myo)fibroblast-targeted therapy holds great promise for suppressing the progression of IPF, its development is hindered by the limited drug delivery efficacy to (myo)fibroblasts and the vicious circle of (myo)fibroblast activation and evasion of apoptosis.

**Methods:** Here, a dual small interfering RNA (siRNA)-loaded delivery system of polymeric micelles is developed to suppress the development of pulmonary fibrosis via a two-arm mechanism. The micelles are endowed with (myo)fibroblast-targeting ability by modifying the Fab' fragment of the anti-platelet-derived growth factor receptor-α (PDGFRα) antibody onto their surface. Two different sequences of siRNA targeting protein tyrosine phosphatase-N13 (PTPN13, a promoter of the resistance of (myo)fibroblasts to Fas-induced apoptosis) and NADPH oxidase-4 (NOX4, a key regulator for (myo)fibroblast differentiation and activation) are loaded into micelles to inhibit the formation of fibroblastic foci.

**Results:** We demonstrate that Fab'-conjugated dual siRNA-micelles exhibit higher affinity to (myo)fibroblasts in fibrotic lung tissue. This Fab'-conjugated dual siRNA-micelle can achieve remarkable antifibrotic effects on the formation of fibroblastic foci by, on the one hand, suppressing (myo)fibroblast activation via siRNA-induced knockdown of NOX4 and, on the other hand, sensitizing (myo)fibroblasts to Fas-induced apoptosis by siRNA-mediated PTPN13 silencing. In addition, this (myo)fibroblast-targeting siRNA-loaded micelle did not induce significant damage to major organs, and no histopathological abnormities were observed in murine models.

**Conclusion:** The (myo)fibroblast-targeting dual siRNA-loaded micelles offer a potential strategy with promising prospects in molecular-targeted fibrosis therapy.

## Introduction

Idiopathic pulmonary fibrosis (IPF) is a relentlessly progressive and inevitably fatal lung disease that is characterized by an unrestrained accumulation of activated fibroblasts and myofibroblasts - which we collectively refer to as (myo)fibroblasts within fibroblastic foci, producing an excess of extracellular matrix (ECM) components 1, 2. Although our understanding of the pathogenic mechanisms of pulmonary fibrosis has advanced greatly in recent years 3, 4, the cause of IPF remains unknown. Genetic determinants 5, aging 6, and environmental exposures 7, including viral infections 8, have been identified as risk factors for this disease. IPF exhibits a poor prognosis, with a median survival time of 3-5 years after diagnosis in the absence of lung transplantation 9, 10. The currently employed pirfenidone and nintedanib are palliative and merely delay disease progression 11, 12, which illustrates the need for an effective treatment for IPF.

Although the mechanisms and progressive nature of IPF are not understood thoroughly, overwhelming pieces of evidence have demonstrated that (myo)fibroblasts are the ultimate culprits responsible for the synthesis and production of ECM components within alveolar structures 13, 14. (Myo)fibroblasts are highly synthetically active cells that are defined by *de novo* development of stress fibers, enhanced contractility, collagenous ECM secretion and alpha-smooth muscle actin (α-SMA) expression 15. Persistent (myo)fibroblast accumulation leads to the formation of fibroblastic foci, and the replacement of normal alveoli by fibroblastic foci and ECM leading to progressive loss of respiratory function is the core pathological characteristic of IPF. Within this context, elimination of fibroblastic foci is considered to be an attractive strategy to treat patients with established pulmonary fibrosis. However, a multitude of challenges is encountered in inhibiting the accumulation of activated (myo)fibroblasts during pulmonary fibrogenesis. First, the high cellular heterogeneity in the fibrotic lung tissue with about 40 different cell types brings challenges for the specific delivery of drugs into the (myo)fibroblasts 16. Hence, it is necessary to develop a (myo)fibroblast-targeting drug delivery system for pulmonary fibrogenesis therapy. Second, since the formation of fibroblastic foci is a complicated pathophysiological process involving (myo)fibroblast phenotypic transition 17 and evasion of apoptosis by (myo)fibroblasts 18, single gene-targeted therapeutic effects is limited. It is reasonable to speculate that simultaneously targeting apoptosis resistance pathways and pathways that contribute to the continuous activation of (myo)fibroblasts might provide better therapeutic effects.

The death receptor Fas is a key regulator of ligand-induced apoptosis in various cell types 19. It was found that IPF (myo)fibroblasts are largely resistant to Fas-induced apoptosis 20, 21. A recent report demonstrated that this resistance could be mediated by the upregulation of an apoptosis inhibitory Fas-interacting tyrosine phosphatase (PTPN13, also known as FAP-1) 22, emphasizing the potential of PTPN13 as an intervention target for promoting (myo)fibroblast apoptosis during the development of IPF. In addition, it is well known that (myo)fibroblast activation is dependent on transforming growth factor-β1 (TGF-β1) 23, and NADPH oxidase-4 (NOX4) has been identified as a master regulator of TGF-β1-induced (myo)fibroblast activation 24. Given the critical role of PTPN13 and NOX4 in the evasion of apoptosis by (myo)fibroblasts and (myo)fibroblast activation during pulmonary fibrogenesis, respectively, it is reasonable to assume that simultaneous interference with the expression of these two proteins may achieve a better therapeutic effects.

Small interfering RNA (siRNA) technology, as a natural approach to silence gene expression with high specificity, has received significant attention 25. However, developing a safe, efficient, and targetable non-viral siRNA delivery system is very challenging due to the insufficient tissue penetration, short circulation lifetime, and poor circulation stability 26. To surmount these inherent drawbacks, nanocarriers demonstrated significant promise due to their outstanding capabilities of relatively high tissue penetrability, cell-/tissue-specific targeting ability, prolonged blood circulation, and less/acceptable toxicity 27. Cationic polymer-carriers, such as polylysine and polyethylenimine (PEI), are extensively used as non-viral gene delivery vectors due to their potential structural diversity and flexible functionality 28. Moreover, the brush polymer of PEG-PEI with the conjugation of polyethylene glycol (PEG) on PEI exhibits excellent biocompatibility and high gene transfection efficiency, representing a promising platform for specific gene therapy 29.

**Figure 1:**
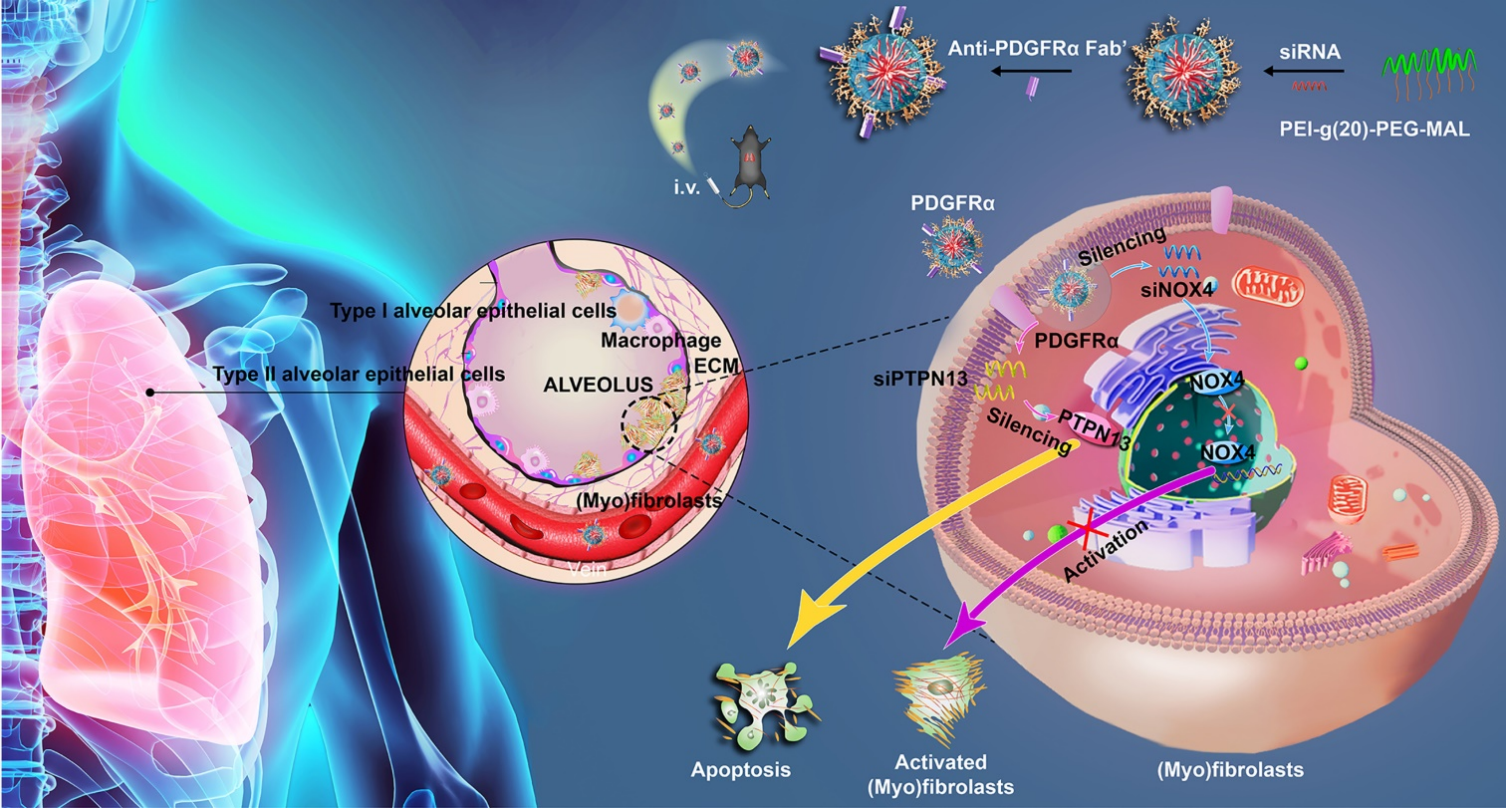
** Schematic illustrating the application of Fab'-conjugated siRNA-loaded micelles for (myo)fibroblast-targeted fibrosis therapy.** The synthesized siRNA-loaded nanocarrier enters the (myo)fibroblasts by targeting to the cellular surface receptor PDGFRα, and then simultaneously promotes (myo)fibroblast apoptosis and inhibits (myo)fibroblast activation.

In this study, a dual-siRNA delivery strategy for IPF therapy *via* (myo)fibroblast-targeting micelles was developed (Figure 1). In particular, the antibody fragment (Fab') of platelet-derived growth factor receptor-α (PDGFRα), which is highly expressed on (myo)fibroblasts, was modified onto the surface of micelles to enhance the (myo)fibroblast-targeted delivery of siRNA. Two different sequences of siRNA that target PTPN13 (a promoter of the resistance of (myo)fibroblasts to Fas-induced apoptosis) 22 and NOX4 (a key regulator of (myo)fibroblast differentiation and activation) 24 were loaded into micelles that were formed by the graft copolymer of branched polyethyleneimine (bPEI) modified with multiple PEGs. Consequently, a significant (myo)fibroblast-targeting effect was achieved in the fibrotic lung tissue of mice administered with the Fab'-conjugated dual siRNA-loaded micelles. In addition, compared with single gene-targeted therapy, simultaneous cotargeting of PTPN13 and NOX4 showed significant antifibrotic effects in the treatment of pulmonary fibrogenesis. This investigation not only enriches the (myo)fibroblast-targeting strategies, but it also offers a promising platform for multigene therapy against pulmonary fibrosis.

## Materials and methods

### Reagents

Bleomycin was purchased from Nippon Kayaku (Tokyo, Japan). Recombinant mouse TGF-β1 was obtained from MedChemExpress (no. HY-P7117, Monmouth Junction, NJ). Recombinant mouse Fas Ligand/TNFSF6 protein was received from R&D Systems (no. 6128-SA-025). Antibodies used in this study are listed in Table S1. Alexa 594-NHS and 3,3'-dithiobis (sulfosuccinimidyl propionate) (DTSSP) fluorescence dyes were purchased from Thermo Fisher Scientific, USA. siRNA for the mouse PNPN13 and NOX4 gene and nonspecific control siRNA were synthesized and purified by GeneChem (Shanghai, China) with the following sequences. PTPN13 siRNA: 5'-GCAGCUAACAGAGACAUUUTT-3'; Scrambled siRNA: 5′-AAUUCUCCGAACGUGUCACGUTT-3′; NOX4 siRNA: 5'-CCAGUGGUUUGC AGAUUUATT-3'; Scrambled siRNA: 5′-UUCUCCGAACGUGUCACGUTT-3′.

### Synthesis of PEI-g(n)-PEG-MAL

The copolymers of PEI-g(n)-PEG-MAL were synthesized as described previously 30. Briefly, 200 mg of PEI (25 kDa) was dissolved in 10 mL of 50 mM phosphate buffer (150 mM sodium chloride, pH 8.0). Two functional PEGs (MAL-PEG5000-NHS and MeO-PEG5000-NHS, at a 1:1 ratio) were added into the PEI solution at a PEG/PEI molar ratio of 20, 30, or 40. The solution was then incubated at room temperature with stirring under nitrogen for 4 h. The remaining free PEG molecules were removed from PEG-grafted PEIs using Vivaspin® 6 (molecular weight cut-off (MWCO) = 10 kDa, 10 mM phosphate-buffered solution at pH 7.4) at 10,000 rpm for four times. The degree of PEG grafting was estimated using the 2,4,6-trinitrobenzene sulfonic acid (TNBS) assay through determining the free primary amine groups that remain on the PEGylated PEIs by following the standard protocol 31. Briefly, 15 μL of 60 mM TNBS was added into each sample (1.1 mL), and the solution was then kept at room temperature for 25 min. TNBS reacts with primary amino groups and produces yellow chromogenic derivatives that could be quantified by measuring their absorbance at 420 nm. Subsequently, 14.7, 19.6, and 23.9 PEG chains were grafted onto each PEI molecule and are referred to as PEI-g(15)-PEG-MAL, PEI-g(20)-PEG-MAL, and PEI-g(24)-PEG-MAL, respectively.

### Preparation of Fab' from antibody

Fab' was generated from the anti-PDGFRα antibody (no.ab203591, Abcam) with a digestion method 32. Briefly, 0.5 mg/mL antibody was digested with 25 μg/mL pepsin dissolved in 0.1 M acetate buffer, pH 4.0, at 37 °C for 8 h. Then the solution pH was adjusted to 7.0 by adding 2 M Tris-base buffer, pH 8.5, and then the solution underwent an ultrafiltration process (MWCO = 50 kDa, 10 mM PBS buffer, pH 7.4) to obtain F(ab')_2_ with pepsin removed and Fc disrupted. DTT (500 μM) was added to 0.5 mg/mL F(ab')_2_ solution with stirring for 30 min at 37 °C to disrupt the disulfide bond in F(ab')_2_. The thus obtained Fab' was purified with a Vivaspin 6 (MWCO = 30 kDa, three times, 10 mM PBS buffer, pH 7.4).

### Preparation of siRNA-loaded micelle

PEI-g-PEG-maleimide (0.2 mg/mL, Figure S1) was mixed with the siRNA solution at different molar ratios of amino/negative charges (3:1, 6:1, and 10:1) in 4-(2-hydroxyethyl)-1-piperazineethanesulfonic acid (HEPES) buffer (20 mM, pH 7.4). After slight overloading, DTSSP was added into the above solution at a DTSSP/NH_2_ ratio of 0, 0.2, 0.4, or 0.8 to encapsulate the siRNA into the micelle core based on the disulfide cross-linking between polymers. The solutions were incubated at room temperature for 25 min and subsequently treated with glycine (10 molar equivalents to DTSSP) for 2 h to quench excess DTSSP. The ratio of conjugated Fab' to micelle was 1.43:1, 2.12:1, or 2.57:1, corresponding to molar ratios of feeding Fab' to polymer of 0.5:1, 1:1, and 2:1, respectively, as quantified by fluorescence correlation spectroscopy (FCS). Note that no significant improvement of the conjugated Fab' number was obtained for the feeding Fab' to polymer ratio of 2:1, which may be due to the steric hindrance of modified Fab' on micelles leading to a decrease of conjugation efficiency. We used a molar ratio of feeding Fab' to polymer of 1:1, corresponding to a ratio of conjugated Fab' to micelles of 2.12, in the following work. The anti-PDGFRα Fab' was then added to the solution at an equal molar ratio to the polymer to conjugate it onto the surface of the nanoparticle. After 4 h of reaction at room temperature, siRNA-loaded micelles were purified with Spectra/Por cellulose ester dialysis membranes (MWCO = 100 kDa, Thomas Scientific, USA).

### Dynamic light scattering and transmission electron microscopy analysis

Dynamic light scattering (DLS) measurements were performed to determine the size distribution of siRNA-encapsulated micelles using a Zetasizer Nano ZS90 (Malvern Instruments Ltd., UK) in PBS buffer (10 mM, pH 7.4) at room temperature. To obtain transmission electron microscopy (TEM) images, 8 μL of micelle solution was placed on a carbon-coated copper grid. The micelle samples were then stained with 2 wt% uranyl acetate, and then the images were taken using a JEM-1400 (JEOL Ltd., Japan).

### Fluorescence correlation spectroscopy

Free Cy3-Fab'- and Fab'-conjugated micelles were separately dissolved in 10 mM PBS (pH 7.4) with or without 10% serum and NaCl (150 mM). FCS measurements were performed using a CLSM 880 equipped with a ConfoCor 3 module (Carl Zeiss, Germany) and a C-Apochromat 40 × water immersion objective. The argon laser was applied for the excitation of Cy3 dye at 514 nm. The diffusion coefficient (D_C_) was calculated from the measured diffusion time normalized to rhodamine 6G (414 μm^2^s^-1^). Then, the particle size in terms of hydrodynamic diameter (DH) was calculated using the following Einstein-Stokes equation:

DH = k_B_T/3_πη_D_C_ (1)

where T is the temperature, k_B_ is the Boltzmann constant, and η is the viscosity of the solution.

### Cell culture

Primary normal mouse pulmonary (myo)fibroblasts were isolated, as previously reported 33. Freshly isolated fibroblasts were cultured at a concentration higher than 10^5^ cells/mL with DMEM/F12 medium (Grand Island, NY, Gibco) containing 15% fetal bovine serum (Gibco) and 1% penicillin and streptomycin, and maintained in a humidified atmosphere of 95% air and 5% CO2 at 37 °C. The cells were passaged at 1:2 using 0.25% trypsin when they reached 70-90% confluence.

### Cellular toxicity of siRNA-loaded micelles

The CCK-8 cell counting kit (no. A331-01, Vazyme, Nanjing, China) was used to analyze the biological effects of siRNA-loaded micelles on the viability of (myo)fibroblasts. Primary mouse pulmonary fibroblasts were treated with TGF-β1 at 2 ng/mL for 48 h to induce (myo)fibroblastic differentiation. Then, the cells were digested and seeded in a 96-well plate at 1.0 × 10^5^ cells per well. After verification of adhesion, cells were incubated with various concentrations of siRNA-loaded micelles (0, 0.5, 2.5, 5, 10, and 15 μg/mL) for 24 h. Next, 10 μL of CCK-8 solution was added to each well, and the cells were subsequently incubated at 37 °C for four h. The absorbance was measured at 450 nm with a multidetection microplate reader (Versamax, Chester, PA).

### Biodistribution analysis of siRNA-loaded micelles

To analyze the biodistribution of delivered siRNA *in vivo*, major organs (liver, spleen, heart, kidney, and lung) were harvested from mice 24 h after injecting 200 μL of Alexa 647-labeled siRNA-loaded micelles. The accumulation of Fab fraction was evaluated by fluorescence measurements using an Infinite M1000 PRO spectrophotometer (Tecan Group Ltd., Männedorf, Switzerland).

### Bleomycin-induced pulmonary fibrosis and treatment

Different animal models have been employed to investigate potential therapies for IPF. Despite its limitation, the bleomycin animal model remains the best available experimental tool for elucidating the pathogenesis of IPF and assessing the efficacy of novel pharmaceutical compounds 34. To establish a bleomycin-induced mouse pulmonary fibrosis model, male C57BL/6 mice (6-7 weeks old) were maintained under specific pathogen-free conditions with free access to water and laboratory rodent chow. Following anesthesia with pentobarbital sodium (3 mg/kg), mice received a single and slow intratracheal injection of bleomycin (2.5 U/kg) dissolved in 50 μL of saline with a MicroSprayer (Penn-Century, Wyndmoor, PA, USA). Control mice received 50 μL of saline only. Seven days after administration of bleomycin, the mice were intravenously injected with siRNA-loaded micelles or Fab' siRNA-loaded micelles every four days. Mice were killed on day 21 after bleomycin instillation.

### Quantitative real-time polymerase chain reaction (qRT-PCR)

Total RNA was extracted from the cultured cells or lung tissues using TRizol reagent (no. R401-01, Vazyme) according to the manufacturer's instructions. The sequences of primer pairs used in this assay are shown in Table S2. qRT-PCR was performed using the SYBR Green qRT-PCR kit (no. Q111-02, Vazyme) on an ABI ViiA 7 Real-Time PCR System (Applied Biosystems, Waltham, MA). The C_t_ values were analyzed using the ΔΔC_t_ method, and the relative quantification of the expression of the target genes was measured using glyceraldehyde-3-phosphate dehydrogenase (GAPDH) mRNA as an internal control.

### Flow cytometric analysis

Cell apoptosis was analyzed by an Annexin V-FITC and PI staining kit (no. A211-01, Vazyme) by following the manufacturer's instructions. Flow cytometry was performed on a FACS CaliburTM flow cytometer, and the data were analyzed using FlowJo software (FlowJo, Ashland, OR, USA).

### Western blot

Proteins were purified from either (myo)fibroblasts or lung tissue. Western blot analysis was performed as previously described 35. Briefly, proteins were separated by SDS-PAGE and then electrophoretically transferred to polyvinylidene difluoride membranes. Then, membranes were blocked with 5% non-fat milk and incubated with specific primary antibodies, including mouse anti-α-smooth muscle actin (α-SMA), rabbit anti-collagen I, rabbit anti-PTPN13, rabbit anti-NOX4, and rabbit anti-GAPDH. Species-matched horseradish peroxidase-conjugated IgG was used as the secondary antibody. Immunoreactive protein bands were detected using an Odyssey Scanning System (LI-COR, Lincoln, NE, USA).

### Immunofluorescence analysis

Immunofluorescence analysis of cells and lung tissues was performed as described previously 35. For this, the following primary antibodies were employed: rat anti-PDGFRα, mouse anti-α-SMA, rabbit anti-PDGFRα, rabbit anti-PTPN13, and rabbit anti-NOX4. Alexa Fluor 488-conjugated goat anti-mouse antibody and Alexa Fluor 594-conjugated goat anti-rabbit antibody (Invitrogen no. A-11001 and A-11037, Carlsbad, CA, 1:200 dilution) were used as secondary antibodies. Nuclei were stained with DAPI (Sigma no. D9542). The images were observed under an FV3000 confocal laser scanning microscope (Olympus, Tokyo, Japan). For quantification analysis, five high-power fields were analyzed in lung sections taken from each mouse. We then determined the percentages of PDGFRα^+^, PTPN13^+^, and NOX4^+^ cells in total α-SMA^+^ cells.

### Immunohistochemistry and hematoxylin-eosin (H&E) and Masson trichrome staining

Sections (5 μm thick) were deparaffinized with xylene before rehydration in an ethanol gradient. Then, endogenous peroxidase activity was quenched by incubating with 3% H_2_O_2_ for 10 min. Sections were then blocked with 3% bovine serum albumin and incubated with rabbit anti-PDGFRα at 4 °C overnight. The primary antibodies were subsequently detected by incubation with horseradish peroxidase-conjugated secondary antibodies (Boster, Wuhan, China) at 37 °C for one h. The DAB Substrate System (DAKO) was used to reveal the immunohistochemical staining.

### Histology and Ashcroft score

The mouse lungs were fixed with a buffered formalin solution overnight, dehydrated, transparentized, and embedded in paraffin before sectioning into slices of 5 μm thick. The slides were stained with H&E for structured observation or with Masson's trichrome stain to detect collagen deposits according to the instructions given by the manufacturer (KeyGen no. KGA224/KGMST-8004, Nanjing, China). The determination of hydroxyproline content was carried out using a hydroxyproline assay kit by following the manufacturer's instructions (Nanjing Jian Cheng Bioengineering Institute, no. A030-3, Nanjing, China). The degree of pulmonary fibrosis was evaluated by a histopathologist blinded to the experimental groups using the validated semiquantitative Ashcroft method 36, 37. Briefly, using 100× magnification, each of 10 successive fields was visually graded from 0 (normal lung) to 8 (total fibrous obliteration of the area). The mean value of the grades obtained for all of the areas was taken as the visual fibrotic score 38.

### Measurements of pulmonary function

The i-STAT Portable Clinical Analyzer and i-STAT G7+ cartridges (Abbott Point of Care, Chicago, IL) were used. Arterial blood was sampled from mice's left ventricle (n = 6 in each group). It was then introduced into the sample well and allowed to fill by passive movement to the indicated level (80-100 μL). After closing the cap on the model well, the cartridge was inserted into the analyzer. After completing the calibration and analysis cycles successfully, partial arterial oxygen pressure (PaO_2_) values were recorded.

### Statistical analysis

All experiments were replicated three times, and the obtained data were presented as mean ± SD. Statistical analysis was performed using ANOVA-test or student's t-test. A *P-*value of less than 0.05 was considered to be statistically significant. All statistical analysis was performed using SPSS 18.0 (SPSS, Chicago, IL).

## Results

### Platelet-derived growth factor receptor-α was explicitly expressed in (myo)fibroblasts within the fibrotic lung tissues

**Figure 2:**
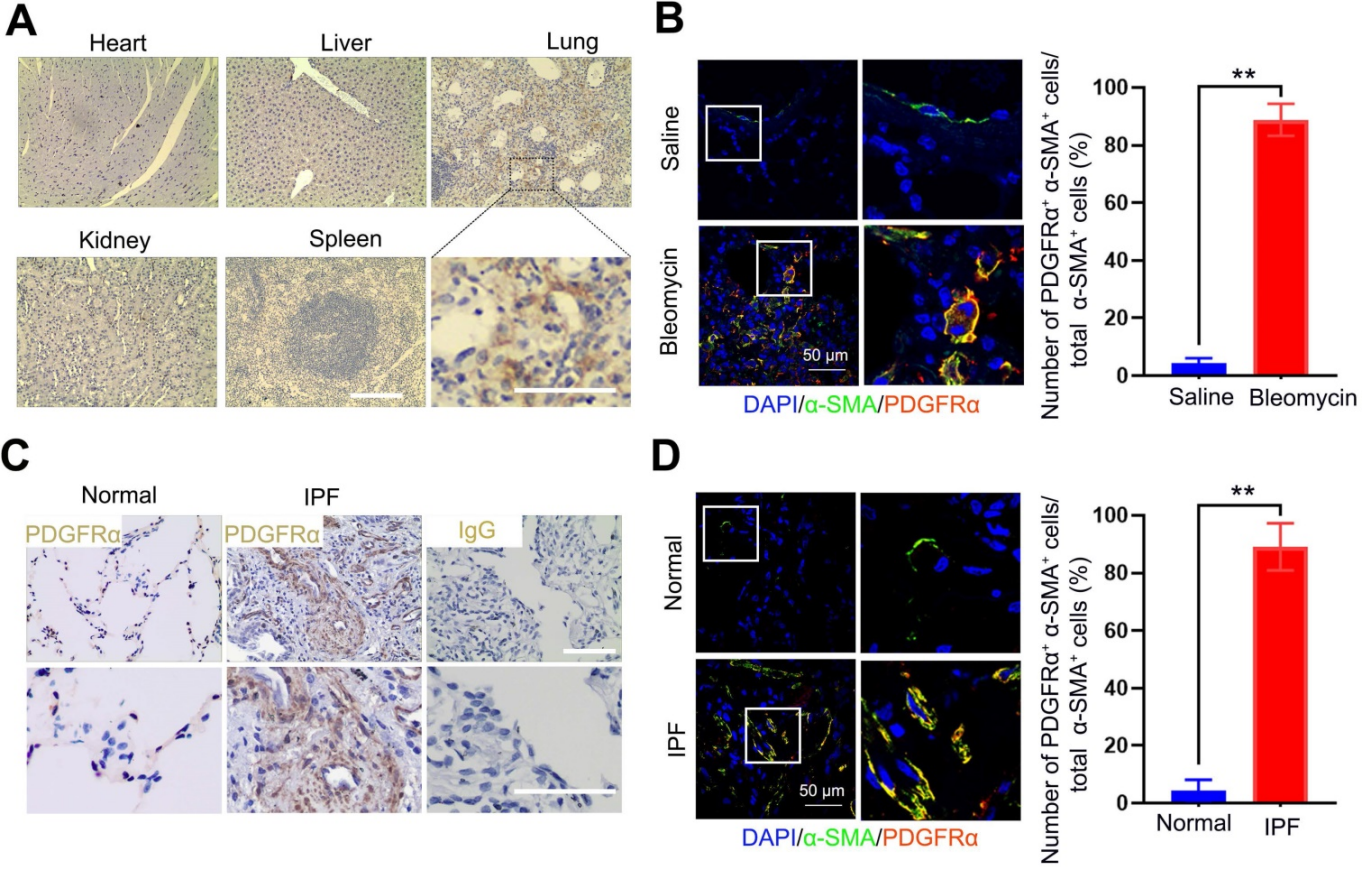
** Distribution and localization of PDGFRα in fibrotic lung tissue and significant organs in bleomycin-treated mice.** (A, B) Mice (n = 5 in each group) received either saline or bleomycin (2.5 U/kg body weight) intratracheally. Mice were sacrificed after 21 days. (A) The expression of PDGFRα in the lung tissues and other organs was examined by immunohistochemistry. Scale bar: 100 µm. (B) The colocalization of PDGFRα and α-SMA in the lung tissues was determined by immunofluorescence assay. Right panels: Quantification of the percentage of PDGFRα^+^ cells in total α-SMA^+^ cells (n = 5, data are expressed as the mean ± SD. **p < 0.01). (C) The expression of PDGFRα in human normal lung tissues (n = 4) and IPF lung tissues (n = 7) was examined by immunohistochemistry. Scale bar: 100 µm. (D) Co-immunofluorescence staining of PDGFRα and α-SMA in human IPF lung tissues. Representative images are shown. Right panels: Quantification of the percentage of PDGFRα^+^ cells in total α-SMA^+^ cells (n = 5, data are expressed as the mean ± SD. **p < 0.01).

Since the expression of PDGFR on (myo)fibroblasts is tissue-specific in different fibrotic diseases, here, we measured the expression of PDGFRα in the lung tissues and other organs bleomycin-treated mice using immunohistochemistry. PDGFRα is abundantly expressed in fibrotic lungs, whereas organs such as the heart, kidneys, liver, and spleen only displayed a weak expression (Figure 2A). Using immunofluorescent staining, PDGFRα was found to localize in (myo)fibroblasts, as evidenced by the colocalization of PDGFRα and the (myo)fibroblast marker α-SMA in the endothelium layer of pulmonary arterioles and microcapillary vessels (Figure 2B). Consistently, there was no prominent expression of PDGFRα in other cells, such as AT Ⅰ cells, AT Ⅱ cells, endothelial cells, and macrophages (Figure S3). Also, these results were confirmed in the lung tissues of IPF patients (Figure 2C-D). These findings demonstrated that PDGFR-α was explicitly present in (myo)fibroblasts within the fibrotic lungs, suggesting its superiority for targeting (myo)fibroblasts *in vivo*.

### The protein levels of PTPN13 and NOX4 were increased upon TGF-β1-induced (myo)fibroblast activation and in fibroblastic foci in fibrotic tissues

**Figure 3:**
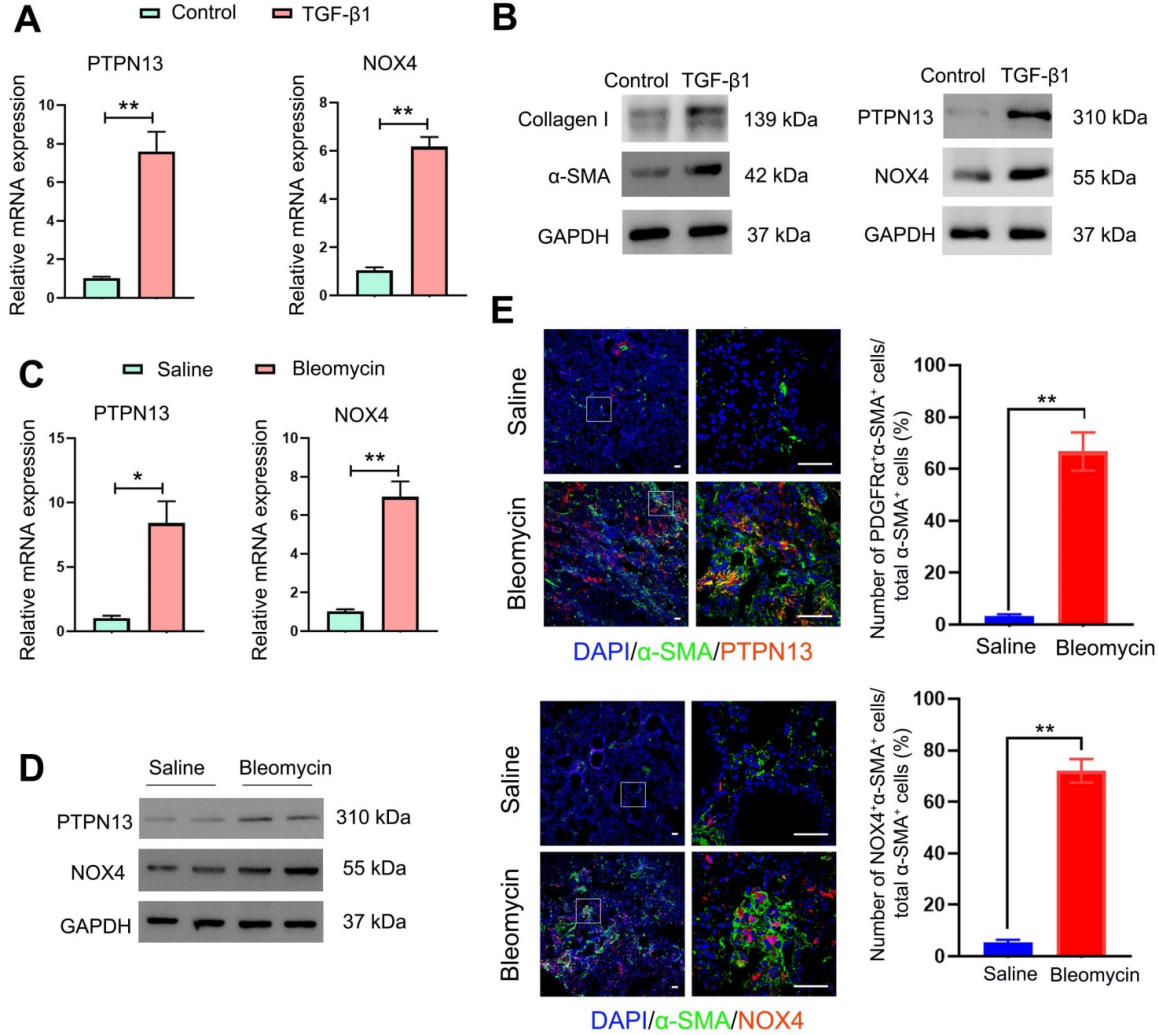
** Overexpression of PTPN13 and NOX4 in activated (myo)fibroblasts and fibrotic lung tissue.** (A-B) Primary normal mouse lung (myo)fibroblasts were stimulated with 2 ng/mL of TGF-β1 for 48 h. (A) The mRNA levels of PTPN13 and NOX4 in the (myo)fibroblasts were examined by qRT-PCR. Data are shown as the mean ± SD (*p < 0.05 vs control group). (B) The expression of collagen I, α-smooth muscle actin (α-SMA), PTPN13, and NOX4 was measured by western blot. (C-E) Mice (n = 5 in each group) received either saline or bleomycin (2.5 U/kg body weight) intratracheally. Mice were sacrificed after 21 days. (C) The mRNA levels of PTPN13 and NOX4 in the lung sections from saline- and bleomycin-treated mice were examined by qRT-PCR. Data are shown as the mean ± SD (*p < 0.05 vs saline group). (D) The western blot technique measured the protein levels of PTPN13 and NOX4. (E) The immunofluorescence assay determined the colocalization of α-SMA ((myo)fibroblast marker) and PTPN13/NOX4. Scale bar: 100 µm. Right panels: Quantification of the percentage of PDGFRα^+^/NOX4^+^ cells in total α-SMA^+^ cells (n = 5, data are expressed as the mean ± SD. **p < 0.01).

Since PTPN13 and NOX4 were identified through their abilities to inhibit Fas-induced apoptosis and to mediate (myo)fibroblast activation, respectively 39, 40, first, the expression levels of PTPN13 and NOX4 were evaluated based on the mRNA levels in fibroblasts treated with TGF-β1, the master regulator of (myo)fibroblast differentiation and activation 41. It was found that both mRNA and protein levels of PTPN13 and NOX4 were significantly elevated, accompanied by an increase in the expression of the (myo)fibroblast marker protein α-SMA (Figure 3A-B). These results were also confirmed in lung tissues derived from the pulmonary fibrosis mouse model (Figure 3C-D). Importantly, these observed data revealed that both PTPN13 and NOX4 were localized to fibroblastic foci in fibrotic lungs, as evidenced by our immunofluorescence assay (Figure 3E). These results indicated that both PTPN13 and NOX4 are closely associated with the formation of fibroblastic foci.

### Preparation and characterization of siRNA-loaded micelles

**Figure 4:**
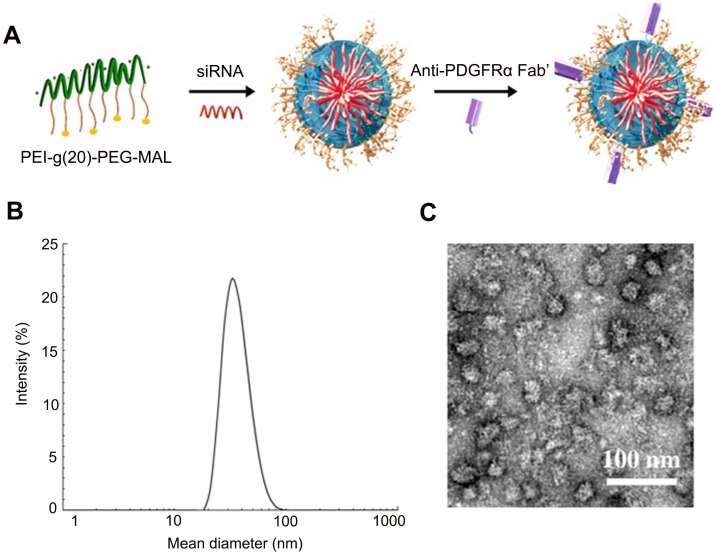
** Formation scheme and characterization of siRNA-loaded micelles.** (A) siRNA-loaded micelles were formed via the assembly of negatively charged siRNA and the positively charged block copolymers PEI-g(20)-PEG-MAL with conjugated Fab' for the targeting of (myo)fibroblasts. Size distribution and morphology images of siRNA (PTPN13)-loaded micelles as measured with DLS (B) and TEM (C). The ratio of N/P is 6:1.

The induction and localization of PDGFRα on the cell membranes of (myo)fibroblasts during pulmonary fibrogenesis prompted us to develop a drug carrier that strongly binds this receptor to achieve homing to the (myo)fibroblasts in the fibrotic lung tissues. To achieve this, PEI-g (n)-PEG-MAL, a traditional siRNA carrier polymer was used to prepare siRNA-loaded micelles. PEI-g (n)-PEG-MAL (n = 15, 20, or 24) polymer was mixed with siRNA at molar ratios of amines in PEI to phosphate groups of RNA bases (N/P) of 3, 6, and 10, respectively, to obtain siRNA-loaded micelles. The polymers of PEI-g(n)-PEG-MAL were used to prepare the siRNA-loaded micelles by mixing with the siRNA solution at N/P ratios of 3:1, 6:1, and 10:1. It was found that the high PEG polymer content of PEI-g(20)-PEG-MAL led to uniform micelle formation with lower PDI (N/P = 6:1, Table S3) than PEI-g(15)-PEG-MAL. The decreased charge density in PEI-g(24)-PEG-MAL due to the higher extent of PEG modification may hamper PM formation stability with siRNA (Table S3). Therefore, we applied the polymer PEI-g(20)-PEG-MAL corresponding to the feeding PEG/PEI ratio of 30 to prepare siRNA-micelles for the subsequent *in vitro* and *in vivo* experiments. Anti-PDGFRα Fab' was conjugated onto the micelles *via* a covalent bond between the thiol group of Fab' and the maleimide group in the polymer PEI-g-PEG-maleimide, aiming to improve the (myo)fibroblasts-targeting efficiency of siRNA-loaded micelles (Figure 4A). The non-conjugated Fab' molecules were then removed with Spectrum Spectra/Por Biotech Cellulose Ester (CE) Dialysis Membrane Tubing (MWCO = 100 kDa, 10 mM PBS, pH 7.4). The ratio of conjugated Fab' to micelle was 2.12:1 as quantified by FCS. Consequently, siPTPN13-loaded nanoparticles (N/P = 6:1) with a mean diameter of 44.5 nm as measured by DLS were obtained (Figure 4B, Table S4). TEM images showed uniform morphology with an average size of 38.3 nm (Figure 4C, Table S4). The siNOX4-loaded nanoparticles (N/P = 6:1) exhibited a similar consistent morphology with a mean diameter of 39.7 nm (TEM, Table S5). Notably, the PDI values for the siRNA (both siPTPN13 and siNOX4)-loaded micelles with N/P = 3:1 and N/P = 10:1 were relatively high (Table S4, S5), and hence the micelles with N/P = 6:1 were chosen for the subsequent *in vitro* and *in vivo* experiments (Table S6, S7). No difference in the size of micelles was noted with different [DTSSP]/[NH_2_] ratios of 0, 0.2, and 0.4 (Table S6). The dimensions of micelles with [DTSSP]/[NH_2_] ratios of 0.6 and 0.8 were abnormally large (Table S6) with high PDI values, which might be due to the formation of particle aggregates mediated by the changes in charges induced by the conjugation of a large amount of DTSSP.

### Fab'-conjugated dual siRNA-loaded micelles inhibited (myo)fibroblast activation and sensitized (myo)fibroblasts to apoptosis *in vitro*

**Figure 5:**
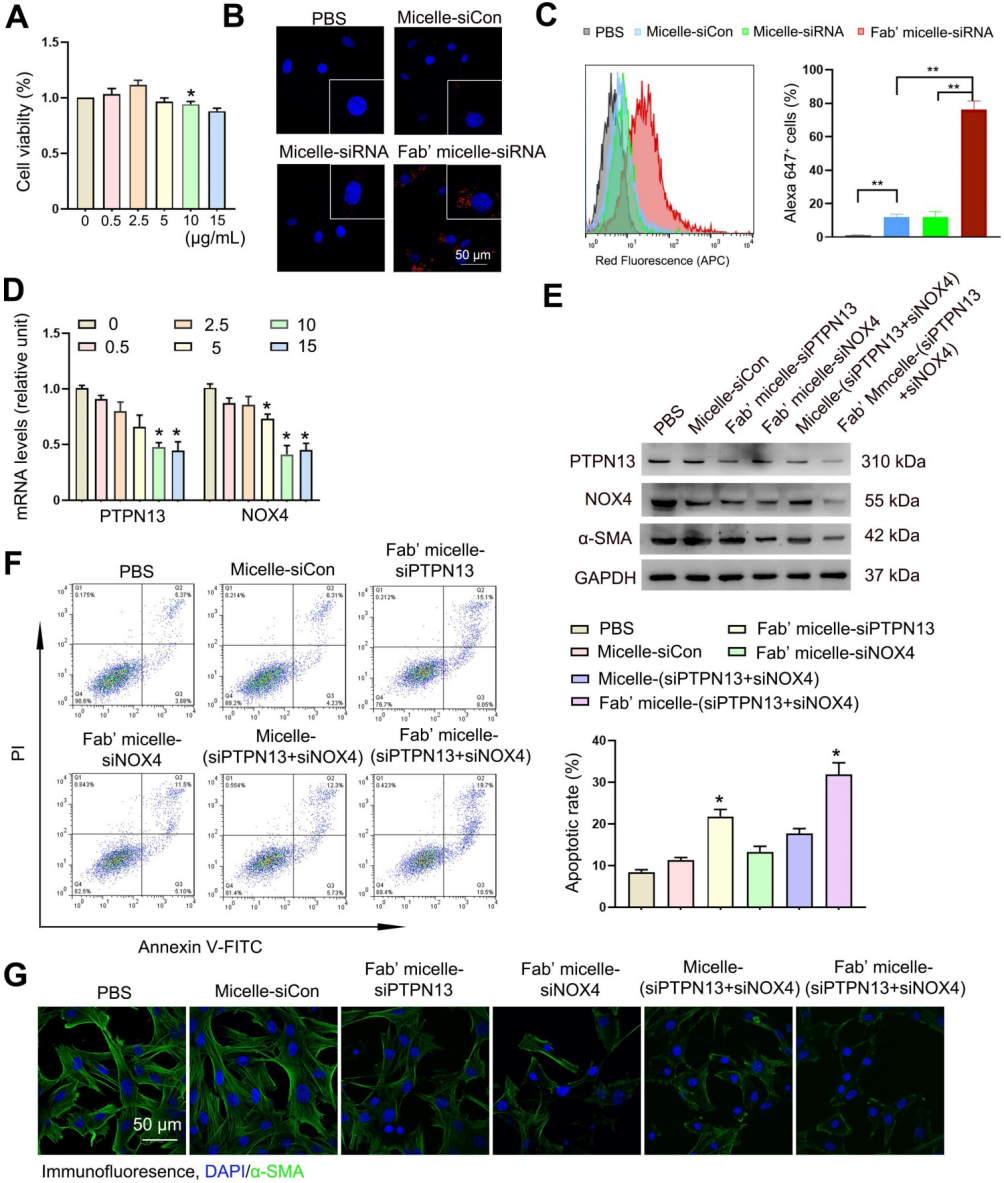
** Efficient delivery of both siPTPN13 and siNOX4 into (myo)fibroblasts by Fab'-conjugated dual siRNA-loaded micelles with potent transfection efficiency *in vitro.*** (A) The viability of (myo)fibroblasts incubated with different concentrations of siRNA and siRNA-loaded micelles for 24 h as measured by the CCK-8 assay. The data are presented as the mean ± SD (*p < 0.05 vs control group). (B) Intracellular distribution of siRNA-loaded micelles in (myo)fibroblasts as determined by immunofluorescence assay. siRNA was labelled with Alexa 647. (C) The percentage of micelle^+^ cells was analyzed by flow cytometry. Rright panels: Quantified data of Alexa 647^+^ cells. Results are expressed as the mean ± SD (**p < 0.01). (D) PTPN13 and NOX4 mRNA levels in (myo)fibroblasts incubated with Fab'-conjugated micelles loaded with different concentrations of siRNA (PTPN13 or NOX4) were measured by qRT-PCR. Data are shown as the mean ± SD (*p < 0.05 compared to the control group). (E) The PTPN13, NOX4, and α-SMA expression levels in (myo)fibroblasts transfected with an siRNA concentration of 5 µg/mL for both free siRNA and Fab'-conjugated siRNA-loaded micelles were determined by western blot. (F) (Myo)fibroblasts were challenged with FasL (100 ng/mL) for 12 h. Apoptosis was evaluated by flow cytometry detecting Annexin V and PI. Results are expressed as the mean ± SD (*p < 0.05 compared with the PBS group). (G) The expression of α-SMA in cells treated with siRNA-loaded micelles.

First, the potential toxicity of PEI-g(20)-PEG-MAL polymer was determined by examining its cytotoxicity at various concentrations, i.e., 0, 0.5, 2.5, 5, 10, 15 μg/mL. The Fab'-conjugated micelles showed low cytotoxicity at these doses after incubation with (myo)fibroblasts for 24 h (Figure 5A).

Next, the cellular uptake of dual siRNA-loaded micelles in (myo)fibroblasts was examined. After 24 h of incubation, (myo)fibroblasts captured a significantly higher amount of Fab'-conjugated micelles than micelles without Fab' conjugation (Figure 5B-C), suggesting the anti-PDGFRα Fab' on the surface of the nanoparticles exerts (myo)fibroblast-targeting effect. Besides, our competition assay demonstrated that the percentage of micelle^+^ cells in the Fab' siRNA-loaded micelle group gradually decreased with increasing concentrations of anti-PDGFRα. These data confirmed the PDGFRα-targeting ability of Fab micelles (Figure S4). Based on the cellular distribution of Fab' conjugated micelles, the Fab'-conjugated dual siRNA-loaded micelles were expected to be effective in simultaneous dual-gene silencing. The *in vitro* dual-gene silencing efficacy of Fab' conjugated micelles was verified by measuring the mRNA levels of PTPN13 and NOX4. The relative expression levels of both PTPN13 and NOX4 showed a substantial decrease in (myo)fibroblasts treated with Fab'-conjugated siRNA-micelles compared to those treated with siRNA-micelles without Fab' conjugation and free siRNA (Figure S5). Also, the mRNA levels of both PTPN13 and NOX4 decreased with increasing doses of siRNA-micelles at concentrations below 10 μg/mL (Figure 5D). As expected, the expression levels of both PTPN13 and NOX4 were decreased, with reduced levels of α-SMA in the cells treated with Fab' conjugated dual siRNA (siPTPN13 and siNOX4)-loaded micelles (Figure 5E). Also, the formation of a protein corona was evaluated by measuring the DH difference of siRNA-micelles in different buffers (HEPES and HEPES with 10% serum included) with an FCS method 42, 43. Almost no difference in DH was found for Fab micelles incubated in HEPES buffer compared with HEPES buffer with 10% serum (Table S8), which indicated that the Fab micelles are stable in serum and do not form a protein corona.

These results confirmed that with their superior (myo)fibroblast-targeting ability, the Fab'-conjugated micelles successfully delivered dual siRNA (siPTPN13 and siNOX4) and achieved excellent knockdown for the cross-linked siRNA-micelles prepared from both siPTPN13 and siNOX4 at N/P_feed_ = 6.0 and [DTSSP]/[NH_2_] = 0.4 by using the equations as shown in Figure S2. These siRNA-micelles were used for the following silencing experiments.

The *in vitro* antifibrotic effects of siRNA-loaded micelles were then further investigated. To determine whether the Fab' conjugated micelles are sufficient to overcome the resistance of (myo)fibroblasts to Fas-induced apoptosis, (myo)fibroblasts were treated with FasL to induce Fas-meditated apoptosis 44. The results revealed that the treatment of Fab'-conjugated micelles remarkably promoted apoptosis in (myo)fibroblasts (Figure 5F). The apoptotic rate of the cells treated with Fab'-conjugated siRNA-micelles was 30.3 ± 1.2%, which is more than double compared to siRNA-micelles without Fab' conjugation and triple compared to siCon-micelles. Moreover, the expression of α-SMA in (myo)fibroblasts treated with Fab' conjugated dual siRNA (siPTPN13 and siNOX4)-loaded micelles was lower than that of Fab'-conjugated siPTPN13 or siNOX4 treatment groups (Figure 5G). These results demonstrated that the ablation of PTPN13 and NOX4 through Fab' conjugated siRNA (PTPN13 and NOX4)-loaded micelles produced a remarkable antifibrotic effect for enhancing the (myo)fibroblast susceptibility to apoptosis and suppressing (myo)fibroblast activation simultaneously. These results also indicated that Fab'-conjugated dual siRNA (siPTPN13 and siNOX4)-loaded micelles have a promising prospect in treating IPF.

### Fab'-conjugated siRNA-loaded micelles efficiently targeted PDGFRα^+^ (myo)fibroblasts in the fibrotic lung tissues

**Figure 6:**
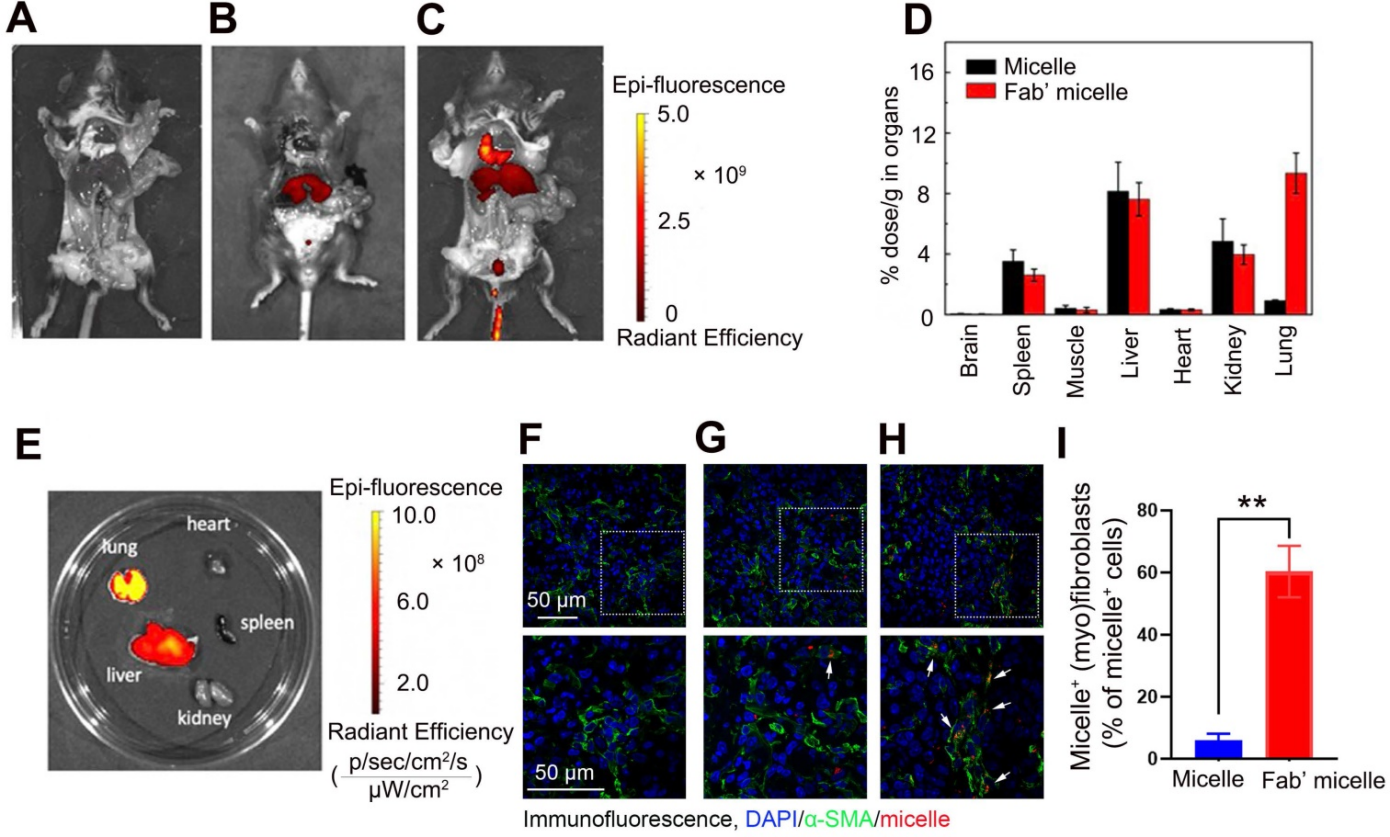
***In vivo* biodistribution of delivered micelles.** (A-C) The Alexa 647 fluorescent images of the C57BL/6 mice and corresponding lung images at 24 h after i.v. injection of 200 µL saline (A), micelles (B) and anti-PDGFRα Fab'-conjugated micelles (C). The biodistribution of administrated siRNA-micelles and anti-PDGFRα Fab'-conjugated micelles in organs (D) and organs' images in mice administered with Fab'-conjugated micelles (E). (F-H) The colocalization of micelle^+^α-SMA^+^ (myo)fibroblasts in the lung tissue of saline- (F), micelle- (G) and anti-PDGFRα Fab'- conjugated micelle-treated (H) mice. (I) Quantified data of micelle^+^ (myo)fibroblasts. Results are expressed as the mean ± SD (n = 5; **p < 0.01 vs. the micelle group). Each mouse was administrated with 3 nmol siRNA loaded micelles. The arrows indicate cells positive for both α-SMA (green) and micelle (red).

To explore the feasibility of targeting (myo)fibroblasts using Fab'-conjugated siRNA-loaded micelles *in vivo*, Fab' conjugated dual siRNA (siPTPN13 and siNOX4)-loaded micelles or siRNA-loaded micelles without Fab' conjugation were injected into the mice one week after the intravenous administration of bleomycin. The fluorescence assay results demonstrated that 24 h after injection, some of the siRNA-loaded micelles without Fab' conjugation accumulate in the lung tissue through blood circulation (Figure 6A-B). Of note, the Fab' conjugated siRNA-loaded micelles exhibited much higher accumulation in the lung tissue than the siRNA-loaded micelles without Fab' moieties (Figure 6B-C), indicating the advantage of the conjugated Fab' directing the nanocarriers to the lung tissue. Besides, the immunofluorescence results demonstrated that the accumulation of Fab' conjugated micelles in lung tissue was significantly higher than that in other visceral organs (Figure 6D-E). The distribution of Fab' conjugated siRNA-loaded micelles in the lung reached ∼9.7% g^-1^, which is about 8.6 times higher than that of the non-targeting micelles (∼1.12% g^-1^) (Figure 6D). We further studied the detailed distribution and location of the micelles inside the lung tissue. The lung tissues' transverse sections were observed by confocal fluorescence microscopy after staining with antibodies (green) against (myo)fibroblast marker α-SMA. The overlapping fluorescence signals of micelles and α-SMA^+^ (myo)fibroblasts (Figure 6F-H) suggested that (myo)fibroblasts internalized the siRNA-loaded micelles. These results explicitly confirmed the advantages of conjugated Fab' in directing the nanocarriers to fibroblastic foci in the lung tissue. Note that the Fab'-conjugated micelles (Figure 6C, 6H, and 6I) exhibited much stronger fluorescence signals in the lung than non-Fab'-conjugated micelles (Figure 6B, 6G, and 6I), which was consistent with the whole animal images. Together, these *in vivo* results demonstrated that the Fab'-conjugated siRNA-loaded micelles could be efficiently delivered to the (myo)fibroblasts.

### The administration of Fab'-conjugated dual siRNA-loaded micelle inhibited the development of bleomycin-induced pulmonary fibrosis

**Figure 7:**
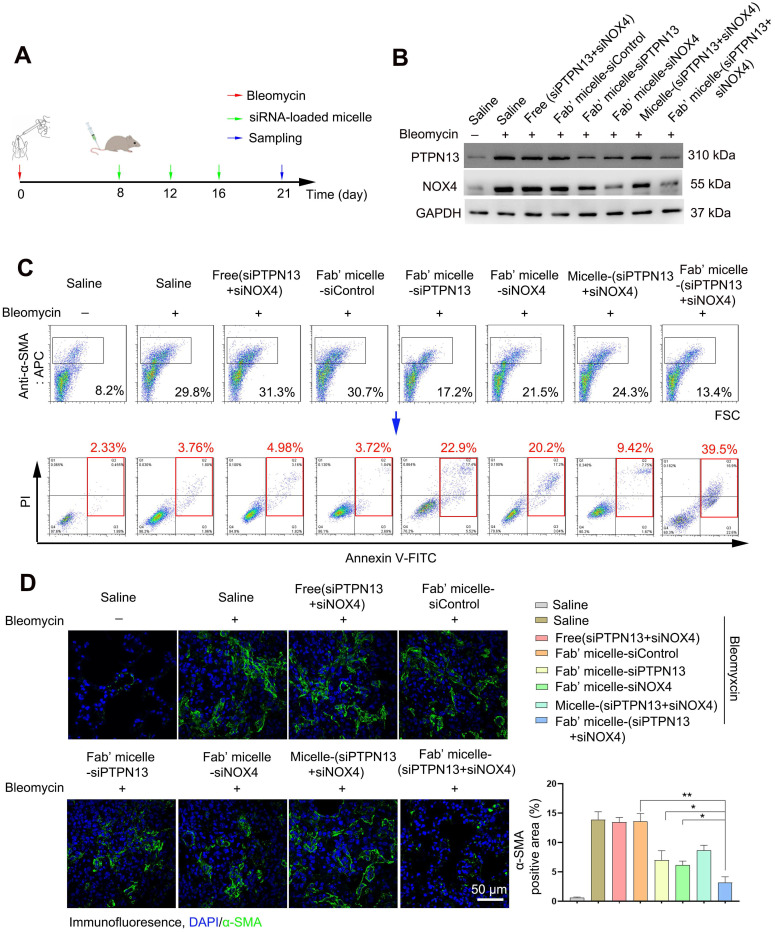
** Apoptosis promoting and (myo)fibroblast activation inhibiting effects of Fab'-conjugated siRNA-loaded micelles on (myo)fibroblasts* in vivo*.** Mice (n = 10 in each group) were intravenously injected with siRNA-loaded micelle or anti-PDGFRα Fab'-conjugated siRNA-loaded micelles 7 days after bleomycin administration. Mice were sacrificed at day 21 after bleomycin instillation. (A) Schematic diagram of the Fab'-conjugated siRNA-loaded micelles therapy procedure. (B) The expression of PTPN13 and NOX4 in lung tissues was measured by western blot. (C) The strategy to identify apoptotic pulmonary (myo)fibroblast subsets from whole lung-digests in bleomycin-treated mice. Apoptotic cells were analyzed by flow cytometry measuring Annexin V and propidium (PI) expression in the α-SMA^+^ cell fraction (n = 3). (D) Immunofluorescence assay examining the expression of α-SMA in the lungs. The positive areas were analyzed by densitometry. Data are shown as the mean ± SD (**P < 0.01, *P < 0.05).

Following the capability of Fab'-conjugated siRNA-loaded micelles for the targeting of (myo)fibroblasts and siRNA delivery, the effect of this siRNA delivery system on bleomycin-induced pulmonary fibrosis was evaluated based on the proposed antifibrotic result of siPTPN13 and siNOX4. Seven days after bleomycin injection, Fab'-conjugated siRNA-loaded micelles were intravenously injected every four days (Figure 7A). The administration of each siRNA (3 nmol) per mouse was performed at a time. As shown in Figure 7B, Fab'-conjugated dual siRNA (siPTPN13 and siNOX4)-loaded micelles successfully reduced the protein expression of both PTPN13 and NOX4, providing substantial evidence of successful target gene silencing. Concordant with our results *in vitro*, we found that treatment with Fab'-conjugated dual siRNA (siPTPN13 and siNOX4)-loaded micelles sensitized pulmonary (myo)fibroblasts to apoptosis and inhibited (myo)fibroblast activation simultaneously (Figure 7C-D). In particular, Fab'-conjugated dual siRNA (siPTPN13 and siNOX4)-loaded micelles were more effective in promoting apoptosis and inhibiting (myo)fibroblast activation, compared to Fab'-single siRNA (PTPN13 or NOX4)-loaded micelles (Figure 7C-D). These data indicated that the simultaneous gene silencing with dual-siRNA achieve therapeutic efficacy on pulmonary fibrosis through impeding the interaction between and cooperation of PTPN13 and NOX4 in fibrosis development.

**Figure 8:**
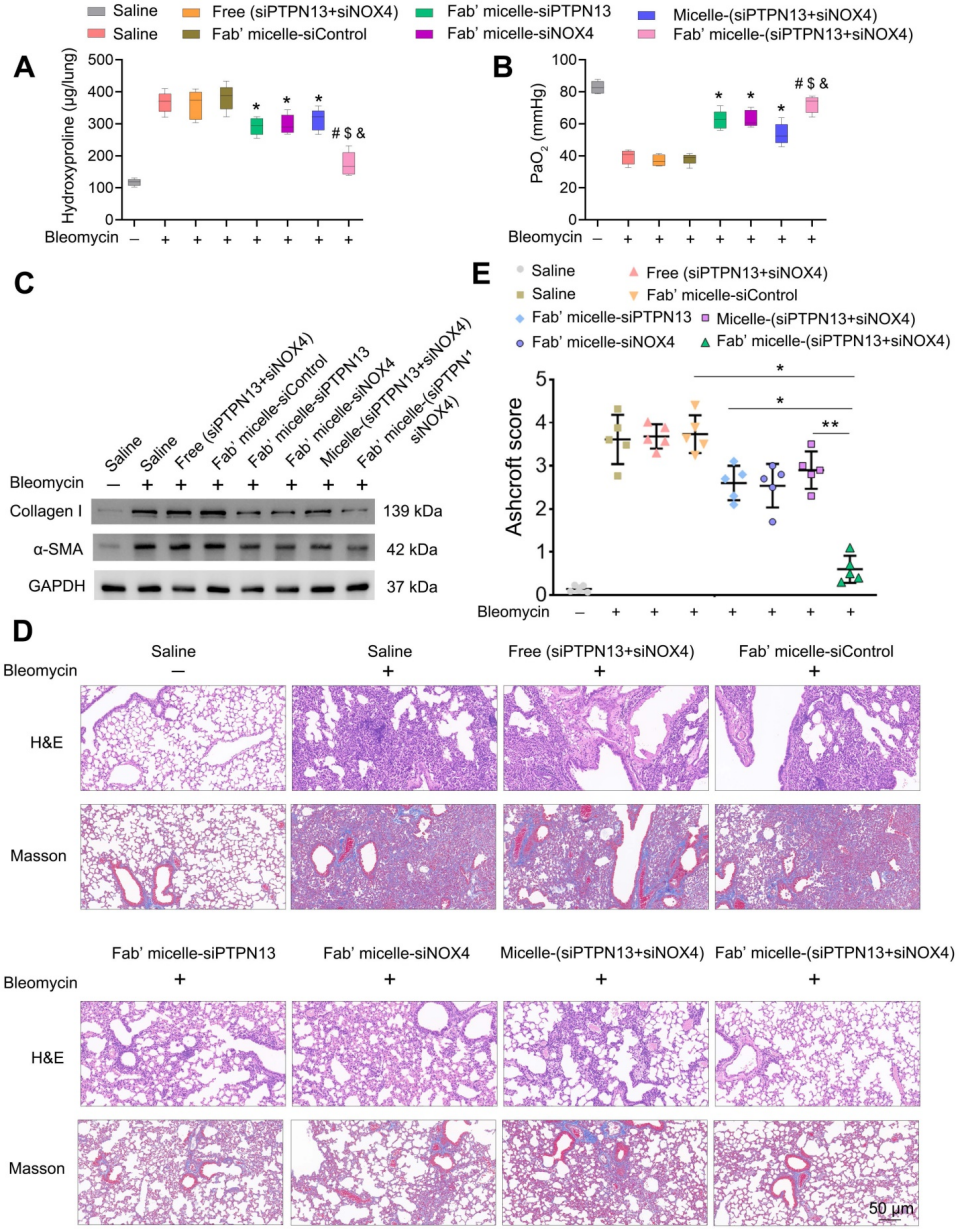
** The therapeutic effects of Fab'-conjugated siRNA-loaded micelles on bleomycin-induced pulmonary fibrosis.** Mice (n = 10 in each group) were intravenously injected with siRNA-loaded micelle or anti-PDGFRα Fab'-conjugated siRNA-loaded micelles 7 days after bleomycin administration. Mice were sacrificed on day 21 after bleomycin instillation. (A-B) The content of hydroxyproline (HYP) and the partial pressure of artery (PaO2) were evaluated through HYP assays (A) and blood gas analysis (B). Values are expressed as the mean ± SD, n = 6. *p < 0.05 compared with the saline group (bleomycin -); #p < 0.05 compared with the saline group (bleomycin +); $ p < 0.05 compared with the Fab'-conjugated siPTPN13-loaded micelle group; &p < 0.05 compared with the Fab'-conjugated siNOX4-loaded micelle group. (C) The expression of collagen I and α-SMA in lung tissues were measured by western blot. GAPDH was used as a loading control. (D) Pulmonary fibrosis was determined by H&E staining, and collagen Ⅰ was visualized by Masson trichrome staining. Representative histological micrographs are shown. (E) The quantification of pulmonary fibrosis based on the Ashcroft score. Values are expressed as the mean ± SD (n = 5. *p < 0.05; **p < 0.01).

We further investigated the therapeutic efficacy of Fab'-conjugated dual siRNA (siPTPN13 and siNOX4)-loaded micelles *in vivo*. As expected, the administration of Fab'-conjugated dual siRNA-loaded micelles to bleomycin-treated mice resulted in a significant reduction in collagen deposition, as evidenced by hydroxyproline measurements (Figure 8A). Consistently, the PaO_2_ levels of the IPF model mice administered with Fab'-conjugated dual siRNA (siPTPN13 and siNOX4)-loaded micelles was much higher than that of mice treated with saline or Fab'-conjugated single siRNA (PTPN13 or NOX4)-loaded micelles (Figure 8B). These results indicated the recovery of lung function of mice treated with Fab'-dual siRNA (PTPN13 and NOX4)-loaded micelles. Besides, bleomycin-induced upregulation of α-SMA and collagen I was suppressed by Fab'-conjugated dual siRNA-loaded micelles (Figure 8C). These results were further confirmed by H&E and Masson's trichrome staining of lungs (Figure 8D). The severity of fibrosis, as classified using the Ashcroft score, was significantly reduced in mice treated with Fab'-conjugated dual siRNA-loaded micelles (0.6±0.28) compared to the micelle-(siPTPN13 and siNOX4) group (2.9±0.37, p < 0.01, Figure 8E). Importantly, the fibrosis score in the Fab'-conjugated dual siRNA-loaded micelle treatment group was notably lower than that in the Fab'-conjugated single siRNA (PTPN13 or NOX4)-loaded micelle treatment group (76% and 72% decrease, respectively, Figure 8E), suggesting that the codelivery of dual siRNA (PTPN13 and NOX4) had a better therapeutic effect than single siRNA on the IPF mice.

After the fibrosis inhibition experiment, the *in vivo* biosafety of Fab' conjugated siRNA-loaded micelles was evaluated. The H&E staining images of the organ samples in the Fab'-conjugated single siRNA (PTPN13 or NOX4)-loaded micelles and the Fab'-conjugated dual siRNA (PTPN13 and NOX4)-loaded micelles treated mice showed no histopathological abnormalities or lesions compared with the saline group (Figure S6). These results demonstrated that the administration of Fab'-conjugated dual siRNA (PTPN13 and NOX4)-loaded micelles did not induce significant damage to major organs.

Overall, the currently developed highly-efficient (myo)fibroblast-targeting dual siRNA delivery system promoted (myo)fibroblast apoptosis and inhibited (myo)fibroblast activation simultaneously (Figure 1). Besides, based on the obtained advantages, this dual-siRNA delivery system has potential to be a safe and promising therapeutic approach for the treatment of IPF.

## Discussion

Although recent advances in our understanding of the molecular events that result in the development and progression of IPF 45, 46, there are still no efficient curative clinical strategies to cure this disease. As the fundamental effector cells in pulmonary fibrogenesis, (myo)fibroblasts are regarded as appealing therapeutic targets in the treatment of IPF 47. However, non-targeted systemic therapies to treat (myo)fibroblasts may not be useful as they may exert unwanted side effects in off-target tissues or cells. Therefore, it is highly desirable to identify factors that are exclusively expressed or overexpressed by (myo)fibroblasts during the progression of IPF, thereby facilitating the development of (myo)fibroblast-targeting treatment strategies.

PDGFRs (PDGFRα and PDGFRβ) are tyrosine kinase receptors which bind to the members of the PDGF family of growth factors 48. Earlier studies suggested that the PDGF/PDGFR axis plays a critical role in tissue homeostasis and repair 49, 50. Specifically, the expression of PDGFRs on (myo)fibroblasts is tissue-specific in different fibrotic diseases 16. In this context, the utilization of PDGFRs of (myo)fibroblasts for the targeted delivery of drugs to treat organ fibrosis has recently gained considerable attention 51. In this study, PDGFRα has been found to be abundantly and exclusively expressed by (myo)fibroblasts in the fibroblastic foci of both IPF patients and bleomycin-treated mice, which is in agreement with earlier reports 16. Considering the high affinity and selectivity of anti-PDGFRα, a fragment (Fab') of anti-PDGFRα antibody-based non-viral nanomicelles was employed to target PDGFRα^+^ (myo)fibroblasts. Note that the excess DTSSP molecules were neutralized by the added glycines the before Fab' conjugation. Therefore, the DTSSP will not affect the Fab' and the specific targeting of PDGFRα. The biodistribution measurements showed that Fab'-conjugated micelles had higher accumulation in the lung than other organs. Due to their efficient binding and intracellular uptake, application of the currently developed Fab'-conjugated micelles as active (myo)fibroblast-specific targeting vehicles is promising. Note that the dense fibrotic tissues due to the deposition and remodeling of ECM proteins may hinder the even better performance of this cell-specific targeting carrier. The development of a proteolytic-enzyme nanoparticular system (e.g., collagenase nanoparticles) 52 may provide new strategies to overcome this problem.

The formation and accumulation of fibroblastic foci is a complex biological process in which both (myo)fibroblast phenotypic transition 53, 54 and evasion of apoptosis by (myo)fibroblasts 18, 20 are considered to be critical pathological events. NOX4 and PTPN13 are vital proteins in (myo)fibroblast activation 24 and resistance of (myo)fibroblast to apoptosis 22, respectively. We speculate that simultaneous suppression of the expression of NOX4 and PTPN13 in (myo)fibroblasts through Fab' conjugated micelles might result in a better therapeutic effect.

siRNA technology has the ability to silence a specific gene and has been broadly exploited in fibrosis's therapeutic development 55-57. Despite its immense potential, gene therapy based on siRNA technology is hindered by inefficient delivery strategies, for example, degradation and off-target effects 26, 58. PEI, a polymer with abundant cationic charges, is an ideal vehicle to deliver siRNA owing to its highly efficient transfection capabilities 59, 60. However, the cytotoxicity of PEI-based nanocarriers impedes their clinical application. It has been reported that the cytotoxicity of PEI is positively correlated with perturbations in cellular membranes, electric charge, molecular weight, and structure 61. Many strategies have been introduced to reduce cytotoxicity of PEI such as statistical surface modification, control of size and topology, and oligoamine segment conjugation. Conjugation of multiple biocompatible PEG molecules as its side chains is a promising strategy to decrease its cytotoxicity. Herein, hydrophilic copolymers of PEI-g(n)-PEG-MAL were synthesized to prepare the siRNA-loaded micelles. The resulting micelles exhibit negligible cytotoxicity, which is consistent with previous studies 62, 63. The low cytotoxicity of the hydrophilic copolymer-based siRNA-loaded micelles is likely related to the shielding of the surface charge by the PEG segment, which in turn reduces the inherent ability of the polymers to disrupt biological membranes.

Through the current investigation, the capability of Fab'-conjugated dual siRNA-loaded micelles to: (i) effectively deliver siNOX4 and siPTPN13 to α-SMA^+^ (myo)fibroblasts and (ii) significantly decrease the expression of these two target genes was confirmed. While no single marker could reliably identify all myofibroblasts, α-SMA, predominantly expressed at a high level, has been the most widely recognized marker of (myo)fibroblasts 24, 64, 65. In contrast, Sun et al. showed that only a minority of collagen-producing cells coexpress of α-SMA in the fibrotic lung of PDGFRβ-*Cre* mice 66. The reason for this discrepancy may be related to the imprecise nature of recombination driven by PDGFRβ-*Cre*. Besides, the SMA-RFP mice they used cannot fully recapitulate endogenous SMA 67. Animals administered with Fab'-conjugated dual siRNA-loaded micelles: (i) revealed significantly lower hydroxyproline levels, collagen, and fibroblastic foci within lung tissues at 21 days following bleomycin administration and (ii) exhibited suppression of pulmonary fibrogenesis. Also, treatments employing Fab'-conjugated single siRNA-loaded micelles achieved limited therapeutic effects. These results also suggested that (myo)fibroblast activation and evasion of apoptosis by (myo)fibroblasts are relatively independent events, each of which can contribute to the formation of fibroblastic foci and pulmonary fibrogenesis. Still, the coadministration of siNOX4 and siPTPN13 via Fab'-conjugated micelles achieved a remarkable therapeutic effect.

It is also important to point out that the antifibrotic effects of the dual siRNA-loaded micelles may be attributed to other possible mechanisms besides targeting profibrotic (myo)fibroblasts. Of note, it has been shown that NOX4 is involved in the regulation of epithelial cell death during the development of pulmonary fibrosis 68, indicating that the dual siRNA-loaded micelles may exert their antifibrotic effects through reducing the expression of NOX4 in epithelial cells and further inhibiting epithelial cell death. Therefore, optimizing the (myo)fibroblast-targeting ability of the siRNA-loaded micelles is worth performing in the future. Moreover, considering the promising clinical application of our (myo)fibroblast-targeting strategy, more works is needed to lower the siRNA dose and improve their transfection efficacy.

The distinguished antifibrotic effects of the Fab'-conjugated dual siRNA-loaded micelles indicated that NOX4 and PTPN13 might play cooperative roles in developing pulmonary fibrosis. One interesting finding in our study is that silencing NOX4 with Fab'-conjugated siNOX4-loaded micelles decreased the expression of PTPN13, suggesting that NOX4 might contribute to the regulation of the expression of PTPN13. However, the mechanisms underlying the interaction between NOX4 and PTPN13 needs to be clarified in future studies.

## Conclusions

In this study, a superior nanocarrier, Fab'-conjugated dual siRNA-loaded micelles, was developed, and its (myo)fibroblast-targeting abilities and a remarkable therapeutic effect on pulmonary fibrosis were illustrated. This (myo)fibroblast-targeting siRNA-encapsulated nanocarrier solves two vital issues associated with the development of an effective treatment strategy for pulmonary fibrosis. One is the cell-specific delivery of therapeutic agents to (myo)fibroblasts, and the other is related to the problem of breaking the vicious circle of (myo)fibroblast activation and evasion of apoptosis. Overall, this study improves the existing (myo)fibroblast-targeting approaches besides widening the spectrum of treatment strategies for fibrotic diseases.

## Supplementary Material

Supplementary figures and tables.

## Abbreviations

BLM: bleomycin
IPF: idiopathic pulmonary fibrosis
NOX4: NADPH oxidase-4
PDGFRα: platelet-derived growth factor receptor α
PTPN13: protein tyrosine phosphatase-N13
