# Co-delivery of siPTPN13 and siNOX4 *via* (myo)fibroblast-targeting polymeric micelles for idiopathic pulmonary fibrosis therapy: Erratum

**DOI:** 10.7150/thno.137233

**Published:** 2026-08-03

**Authors:** Jiwei Hou, Qijian Ji, Jie Ji, Shenghong Ju, Chun Xu, Xueqing Yong, Xiaoxuan Xu, Mohd. Muddassir, Xiang Chen, Jinbing Xie, Xiaodong Han

**Affiliations:** 1 Immunology and Reproduction Biology Laboratory & State Key Laboratory of Analytical Chemistry for Life Science, Medical School, Nanjing University, Nanjing, 210093, China.; 2 Jiangsu Key Laboratory of Molecular Medicine, Nanjing University, Nanjing, 210093, China.; 3 Department of Critical Care Medicine, Xuyi People's Hospital, 28 Hongwu Road, Xuyi, 211700, Jiangsu, China.; 4 Department of Emergency Medicine, Jinling Hospital, Medical School of Nanjing University, Nanjing, 210002, PR China.; 5 Jiangsu Key Laboratory of Molecular Imaging and Functional Imaging, Department of Radiology, Zhongda Hospital, Medical School, Southeast University, 87 Dingjiaqiao Road, Nanjing 210009, China.; 6 Department of Pathology, Medical School of Southeast University, 87 Dingjiaqiao, Nanjing, 210009, China.; 7 Department of Nuclear Science & Technology, Nanjing University of Aeronautics and Astronautics, Nanjing, 211106, China.; 8 Department of Chemistry, College of Science, King Saud University, Riyadh 11451, Saudi Arabia.

The authors regret that the originally published version of this article contained errors in Figure 3D and Figure 5G. First, an incorrect GAPDH blot was inadvertently used in Figure 3D. The correct version of Figure 3D is shown below. In addition, an incorrect image in Figure 5G was inadvertently included due to an oversight during the final figure assembly. The correct version of Figure 5G is also shown below. All authors concur with these corrections and confirm that they do not alter the scientific findings, interpretations, or the overall conclusions of the study.

The authors apologize for any inconvenience that these errors may have caused.

## Figures and Tables

**Figure A:**
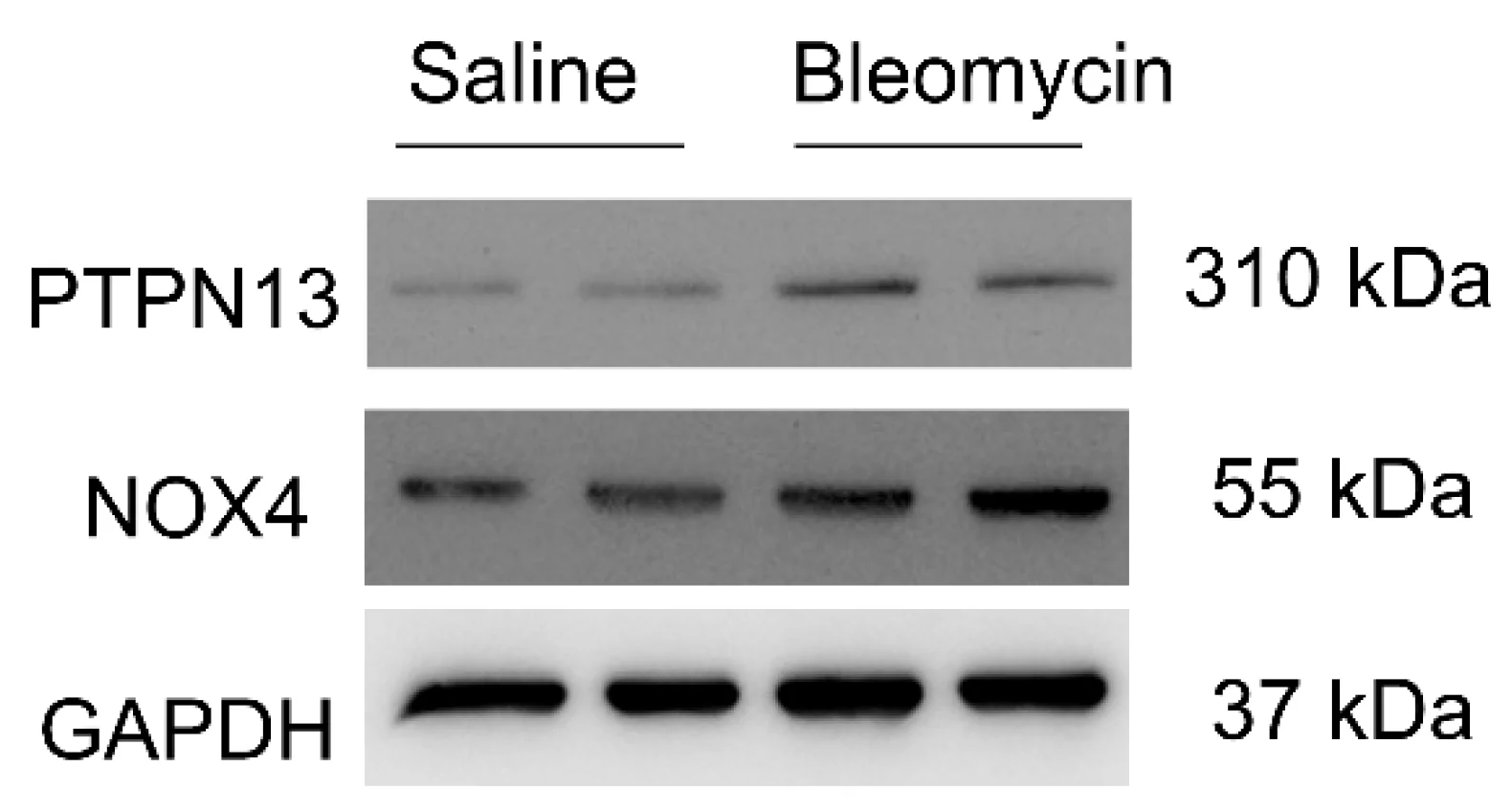
** Corrected figure for original figure 3D.** The western blot technique measured the protein levels of PTPN13 and NOX4.

**Figure B:**
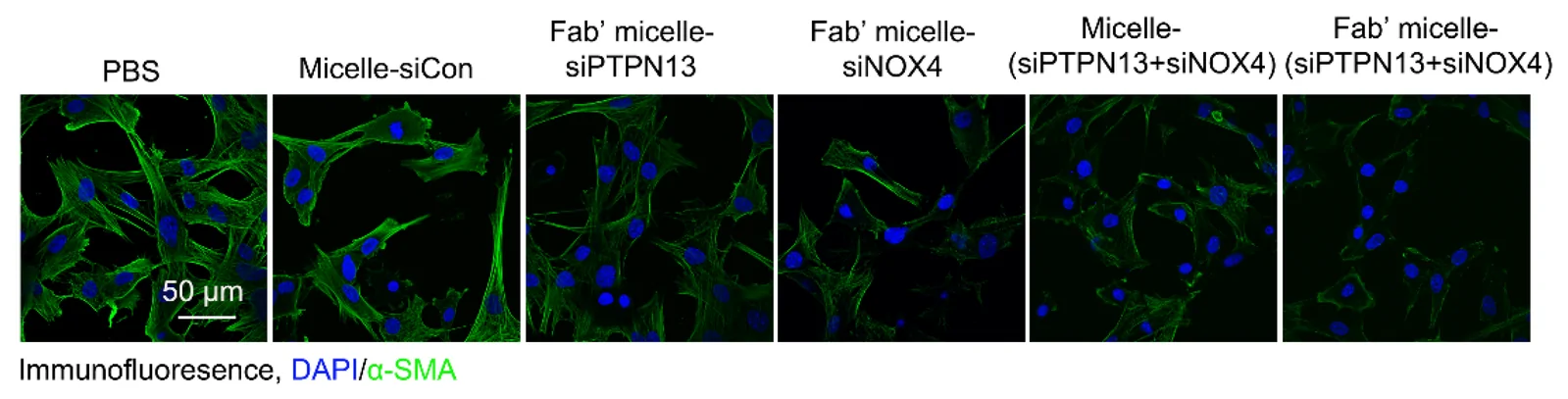
** Corrected Figure for original figure 5G.** The expression of α-SMA in cells treated with siRNA-loaded micelles.

