# Supplementary material for: Co-delivery of siPTPN13 and siNOX4 via (myo)fibroblast-targeting polymeric micelles for idiopathic pulmonary fibrosis therapy
Source: Theranostics. 2021 Jan 9;11(7):3244–61. doi: 10.7150/thno.54217 (PMC7847691; doi:10.7150/thno.54217)
Supplement: Supplementary file 1 — Supplementary figures and tables. [file thnov11p3244s1.pdf]

Co-delivery of siPTPN13 and siNOX4 *via* (myo)fibroblast-targeting polymeric micelles for idiopathic pulmonary fibrosis therapy

Jiwei Hou[1,2,#], Qijian Ji[3,4,#], Jie Ji[1,2], Shenghong Ju[5, *], Chun Xu[6], Xueqing Yong[7], Xiaoxuan Xu[5]， Mohd. Muddassir[8], Xiang Chen[1,2], Jinbing Xie[5,*], Xiaodong Han[1,2,*]

[1] Immunology and Reproduction Biology Laboratory & State Key Laboratory of Analytical Chemistry for Life Science, Medical School, Nanjing University, Nanjing, 210093, China.

[2] Jiangsu Key Laboratory of Molecular Medicine, Nanjing University, Nanjing, 210093, China

[3] Department of Critical Care Medicine, Xuyi People's Hospital, 28 Hongwu Road, Xuyi, 211700, Jiangsu, China.

[4] Department of Emergency Medicine, Jinling Hospital, Medical School of Nanjing University, Nanjing, 210002, PR China.

[5] Jiangsu Key Laboratory of Molecular Imaging and Functional Imaging, Department of Radiology, Zhongda Hospital, Medical School, Southeast University, 87 Dingjiaqiao Road, Nanjing 210009, China.

[6] Department of Pathology, Medical School of Southeast University, 87 Dingjiaqiao, Nanjing, 210009, China.

[7] Department of Nuclear Science & Technology, Nanjing University of Aeronautics and Astronautics, Nanjing, 211106, China.

[8] Department of Chemistry, College of Science, King Saud University, Riyadh 11451, Saudi Arabia

[#]These authors contributed equally: Jiwei Hou, Qijian Ji

*Corresponding authors: Sheng-hong Ju (jsh0836@hotmail.com), Jinbing Xie (xiejb@seu.edu.cn) or Xiaodong Han (hanxd@nju.edu.cn)

Table S1. Specifications of primary antibodies

Vendor	Antibody	Catalog no.	Working dilution
Cell Signaling	Rabbit anti-PDGF Receptor α	#3174	WB, 1:1000 IF, 1:500 IHC, 1:500
ABclonal	Rabbit anti-PTPN13	A13005	WB, 1:1000 IF, 1:100 IHC, 1:100
	Rabbit anti-CD31	A4900	IF, 1:100
Abcam	Rabbit Anti-α-SMA (APC)	ab223921	Flow Cyt, 1:200
	Rabbit anti-SP-C	ab211326	IF, 1:150
Santa cruz	Mouse anti-AQP5	sc-514022	IF, 1:50
	Mouse anti- PDGFRα	sc-398206	IF, 1:50
Bioss	Rabbit anti-F4/80	bs-11182R	IF, 1:50
Boster	Rabbit anti-NOX4	BM4135	WB, 1:1000 IF, 1:50 IHC, 1:50
	Rabbit anti-Collagen I	BA0325	WB, 1:1000
	Mouse anti-α-SMA	BM0002	WB, 1:1000 IF, 1:50
	Mouse anti-GAPDH	BM3876	WB, 1:5000

Table S2. Primers used for real-time PCR.

Accession Number	Target gene	Primer sequence (5'-3') Formard	Reverse	Size (bp)	Tm (℃)
NM_007392.3	α-SMA	CAGCCATCTTTCATTGGGATGGA	TGGTACCCCCTGACAGGAC	124	60
NM_011204.2	PTPN13	CCTAAGGGAAATGGCCCTGG	CCTTCTTGGTCTGCGAGAGG	140	60
NM_001285833.1	NOX4	CCAAATGTTGGGCGATTGTGT	TCCTGCTAGGGACCTTCTGT	133	60
NM _001289726.1	GAPDH	CCCTTAAGAGGGATGCTGCC	TACGGCCAAATCCGTTCACA	124	60

Table S3. The characterization (size, PDI) of siPTPN13-loaded micelles with different polymers at various N/P_{feed} ratio of 3, 6 and 10, respectively.

Polymers	N/P	Size from DLS in PBS (nm)	PDI (PBS) from DLS
PEI-g(15)-PEG-MAL	3	78.8 ± 7.7	0.677
	6	93.3 ± 8.3	0.563
	10	88.4 ± 7.9	0.576
PEI-g(20)-PEG-MAL	3	29.8 ± 1.7	0.207
	6	44.5 ± 2.9	0.137
	10	68.8 ± 7.4	0.306
PEI-g(24)-PEG-MAL	3	128.5 ± 8.3	0.728
	6	175.6 ± 7.8	0.672
	10	145.7 ± 8.4	0.623

Table S4. The characterization (size, PDI, and Zeta potential) of siPTPN13-loaded micelles with various ratio of N/P_{feed}. $[DTSSP]/[NH_2] = 0.4$, n = 5.

PIC micelles	N/P	Mean size from TEM (n=50, nm)	Size from DLS in PBS (nm)	PDI (PBS) from DLS	Zeta Potential (mV)
Micelle-siPTPN13	3	21.5 ± 0.77	29.8 ± 1.7	0.207	2.6 ± 0.8
Micelle-siPTPN13	6	36.4 ± 2.8	44.5 ± 2.9	0.137	7.5 ± 0.7
Micelle-siPTPN13	10	59.7 ± 5.5	68.8 ± 7.4	0.306	13.6 ± 1.8
Fab’ Micelle-siPTPN13	6	38.3±2.5	45.9±3.2	0.175	7.1±0.5

Table S5. The characterization (size, PDI, and Zeta potential) of siNOX4-loaded micelles with various ratio of N/P_{feed}. [DTSSP]/[NH_2] = 0.4, n = 5.

PIC micelles	N/P	Mean size from TEM (n=50, nm)	Size from DLS in PBS (nm)	PDI (PBS) from DLS	Zeta Potential (mV)
Micelle-siNOX4	3	23.3 ± 0.9	35.8 ± 2.2	0.365	3.3 ± 0.8
Micelle-siNOX4	6	38.5 ± 1.9	46.3 ± 3.2	0.145	6.9 ± 0.7
Micelle-siNOX4	10	58.4 ± 3.2	69.3 ± 5.4	0.344	13.4 ± 1.9
Fab' Micelle-siNOX4	6	39.7 ± 3.9	48.9 ± 2.7	0.165	6.1± 0.6

Table S6. The size and PDI of siRNA-micelles (N/P_{feed} = 6) with various ratio of [DTSSP]/[NH_2] as measured with DLS, n = 5.

Ratio of [DTSSP]/[NH_2]	0	0.2	0.4	0.6	0.8
Micelle-siPTPN13(Size, nm)	41.3 ±1.9	42.7±1.7	44.5±2.9	93.3±5.8	123.5±8.3
Micelle-siPTPN13 (PDI)	0.137	0.142	0.137	0.388	0.476
Micelle-siNOX4(Size, nm)	46.4±1.4	47.8±2.6	46.3±3.2	97.5±4.8	138.4±9.2
Micelle-siNOX4 (PDI)	0.126	0.152	0.145	0.402	0.488

Table S7. The characterization of siRNA-loaded micelles, the ratio of N/P is 6 and $[DTSSP]/[NH_2]$ is 0.4 for all samples (n = 5).

PIC micelles	Mean size from TEM (n=30, nm)	Size from DLS in PBS (nm)	PDI (PBS) from DLS	Zeta Potential (mV)
Micelle-siPTPN13	36.4±2.8	44.5±2.9	0.137	7.5±0.7
Micelle-siNOX4	38.5±1.9	46.3±3.2	0.145	6.9±0.7
Micelle-(siPTPN13+ siNOX4)	37.3±2.6	45.7±1.9	0.137	9.3±0.6
Fab' Micelle-siPTPN13	38.3±2.5	45.9±3.2	0.175	7.1±0.5
Fab' Micelle-siNOX4	39.7±3.9	48.9±2.7	0.165	6.1±0.6
Fab' Micelle-(siPTPN13+ siNOX4)	41.5±2.9	48.2±2.6	0.177	8.4±0.8
Fab' Micelle-siControl	42.7±3.3	49.4±3.1	0.183	8.1±0.3

Table S8. The hydrodynamic diameter (DH) of Fab' siRNA-loaded micelles as measured by FCS. The ratio of N/P is 6 and [DTSSP]/[NH_2] is 0.4 for all samples (n = 5).

PIC micelles	DH in HEPES (nm)	DH in 10% serum included HEPES (nm)
Fab' Micelle-siPTPN13	48.3±3.1	48.9±2.7
Fab' Micelle-siNOX4	46.9±2.8	45.5±2.1
Fab' Micelle-(siPTPN13+ siNOX4)	51.1±2.7	51.4±2.3

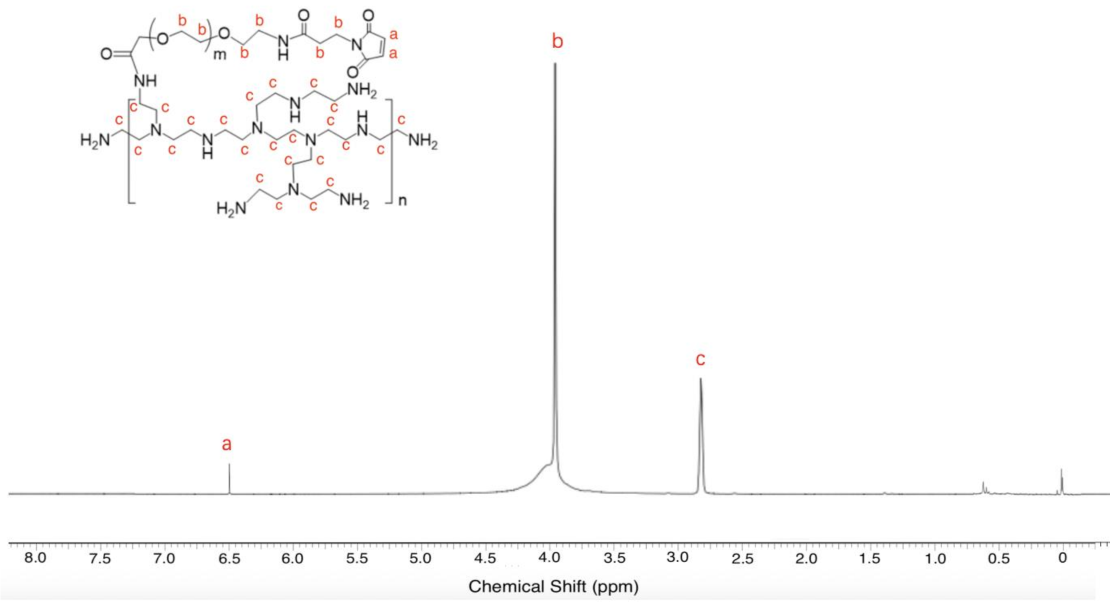

Figure S1. The ^{1}H-NMR spectra of PEI-g(20)-PEG-MAL in D_2O at 25ºC. a: the protons from maleimide (-CH=CH-); b: protons from PEG (-CH2CH2O-); c: the protons from PEI (N-CH2CH2-n).

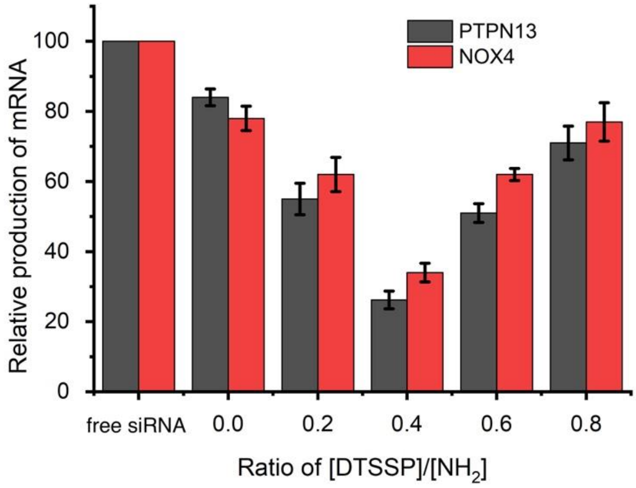

Figure S2. The crosslinking of siRNA-loaded micelles affects the mRNA levels in myofibroblasts. PTPN13 and NOX4 mRNA levels in myofibroblasts incubated with Fab' micelles-(siPTPN13+siNOX4) which was crosslinked with various ratio of [DTSSP]/[NH_2], as measured by qRT-PCR. The ratio of N/P_{feed} is 6:1. Data are shown as the means ± SD.

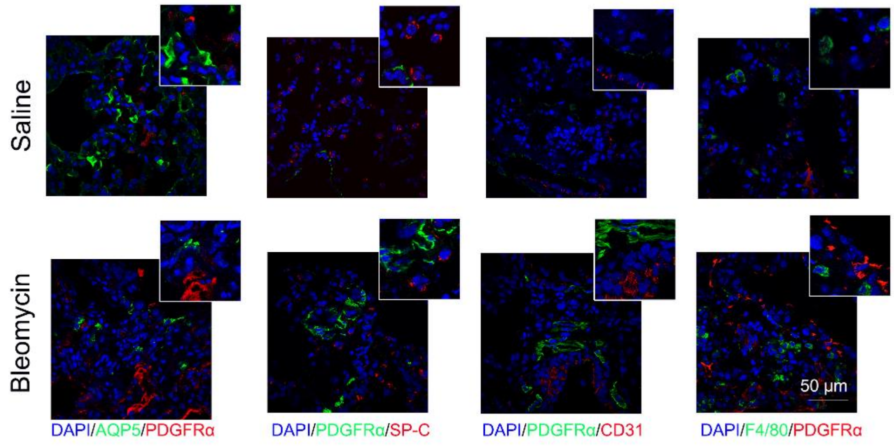

Figure S3. Mice (n = 10 in each group) received either saline or bleomycin (BLM, 5 mg/kg body weight) intratracheally. Mice were sacrificed 21 days later. The colocalization of PDGFRα with AQP5 (AT I cell marker), SP-C (AT II cell marker), CD31 (endothelial cell marker) or F4/80 (macrophage marker) was determined by immunofluorescence assay.

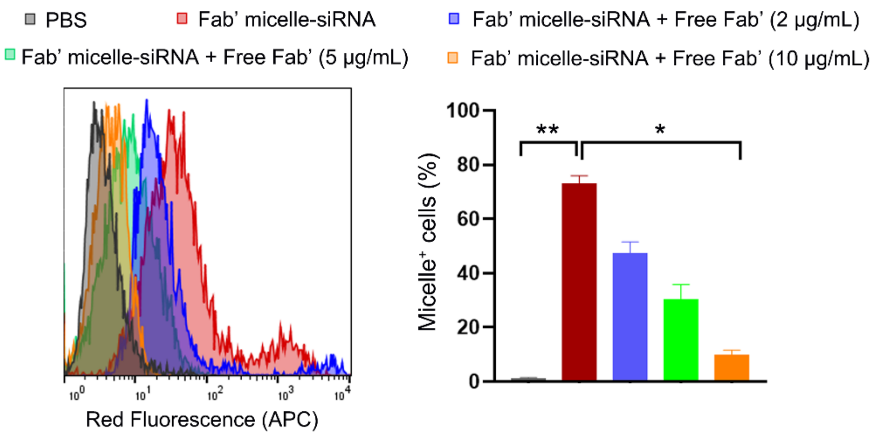

Figure S4: The percentage of micelles-positive cells of Fab' micelle-siRNA treatment group gradually decreased with the increase of concentration of PDGFRα antibody. Right panels: Quantified data of micelle^{+} cells. Results are expressed as means ± SD (n = 5; *p < 0.05, **p < 0.01).

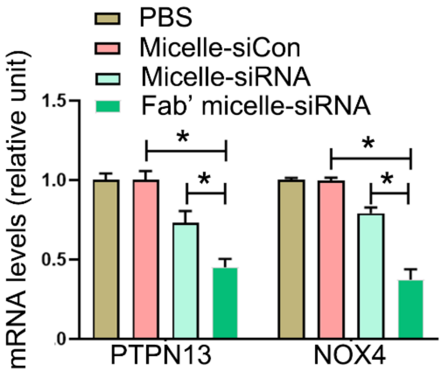

Figure S5: Fab' conjugated dual siRNA (siPTPN13 and siNOX4)-loaded micelles successfully reduced the expression of both PTPN13 and NOX4 *in vitro*. The mRNA levels of PTPN13 and NOX4 in (myo)fibroblasts with the administration of control siRNA, siRNA-loaded micelles with and without conjugation of Fab', respectively, were examined by qRT-PCR. The siRNA concentration was 5 μg/mL for all the samples of control siRNA, siRNA-loaded micelles with and without conjugation of Fab'. Data are shown as means ± SD (*p < 0.05).

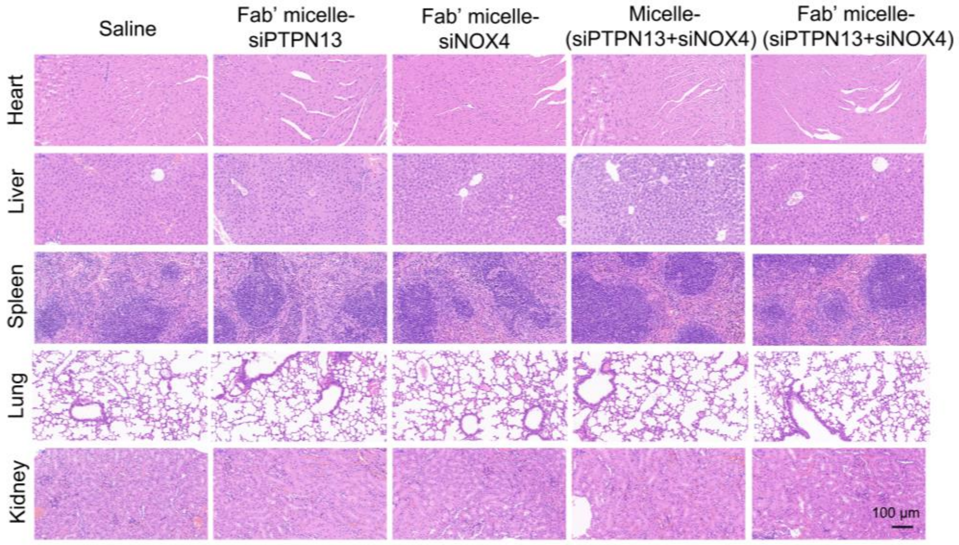

Figure S6. Biosafety evaluation of Fab'-dual siRNA (PTPN13 and NOX4)-loaded micelles *in vivo.* Histopathologic analyses of H&E-stained tissue sections of main organs (heart, liver, spleen, lung, and kidney) of mice after the indicated treatment.
